# Real-time GPU-accelerated coupled cardiac system: Integrating bidirectional interactions between living optogenetic monolayers and computational simulations

**DOI:** 10.1371/journal.pcbi.1014590

**Published:** 2026-08-03

**Authors:** Younes Valibeigi, Abouzar Kaboudian, Flavio Fenton, Gil Bub

**Affiliations:** 1 Department of Physiology, McGill University, Montréal, Quebec, Canada; 2 Montreal Neurological Institute, McGill University, Montréal, Quebec, Canada; 3 School of Physics, Georgia Institute of Technology, Atlanta, Georgia, United States of America; UCI BME: University of California Irvine Department of Biomedical Engineering, UNITED STATES OF AMERICA

## Abstract

**Objective:**

Reentrant arrhythmias are life-threatening cardiac events that are difficult to study due to limited experimental control over the complex circuit dynamics. We present a real-time coupled cardiac system that allows in real-time dynamic manipulation of reentrant pathways in vitro using physiologically relevant simulations.

**Methods:**

We designed a closed-feedback loop system that couples a cultured cardiac monolayer with a two-dimensional computational simulation of cardiac tissue. The simulation, based on GPU-accelerated models (e.g., cellular automata), predicts wave propagation in real-time using the Abubu.js library. Optical mapping captures monolayer activation patterns, and simulation outputs are converted into light-based stimulation via optogenetics, using LEDs and microcontrollers to depolarize cardiac tissue.

**Results:**

Our platform is capable of accurately detecting and responding to electrical waves in real-time, enabling interactive modulation of reentrant circuits. The system replaces traditional fixed-delay stimulation protocols with computationally guided interventions, better mimicking physiological conduction dynamics.

**Conclusion:**

This coupled system provides a novel and responsive method to study reentrant arrhythmias. Its integration of optical stimulation, real-time modeling, and tissue feedback enables the construction of user-defined reentry pathways and dynamic interaction with reentrant circuit behavior.

**Significance:**

By merging computational and biological systems, this work introduces a versatile experimental framework for investigating arrhythmias. Built from inexpensive and accessible components, it lowers technical and financial barriers, increasing accessibility across a broad range of researchers and research environments. The platform may inform future control and anti-arrhythmic strategies and pave the way for personalized cardiac electrophysiology studies.

## Author summary

Irregular heart rhythms caused by reentrant electrical activity can lead to serious and life-threatening conditions. These patterns are difficult to study because they involve complex and rapidly changing waves that are hard to control in traditional experiments. In this work, we developed a system that directly links living heart cells grown in the lab with a computer model capable of simulating electrical activity in real-time. This connection allows the model to both receive information from the cells and deliver targeted stimulation, creating a continuous feedback loop. Unlike conventional approaches that rely on fixed timing, our system adapts dynamically to the behavior of the tissue, enabling real-time modulation of how electrical waves form and propagate. This makes it possible to create, modify, and study reentrant circuits in a controlled and interactive way. Because the platform is built using accessible and low-cost components, it can be implemented in a wide range of research settings. More broadly, this approach provides a new way to study heart rhythm disorders and may help guide the development of personalized and adaptive treatment strategies in the future.

## I. Introduction

**Fig 1:**
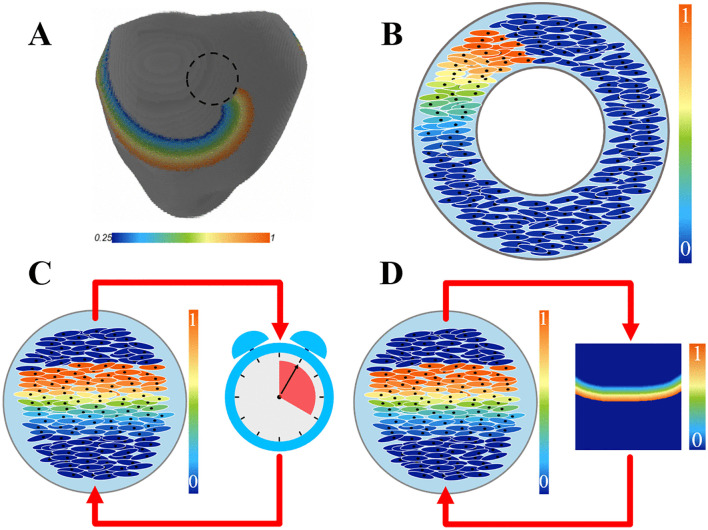
Conceptual overview of the closed-loop coupled cardiac system. **(A)** A 3D cellular automaton model of the heart showing cardiac waves circulating around an inexcitable region (dashed circle), representing a reentrant circuit. **(B)** Schematic of a cardiac monolayer grown around a circular obstacle, illustrating a reentrant circuit with a clockwise-propagating wave around the center; the colorbar indicates cellular voltage. **(C)** Closed-loop system with a fixed delay applied to a cardiac monolayer exhibiting linear wave propagation. **(D)** Full implementation of the coupled system, where real-time signals from the tissue update the computational model, and model predictions drive adaptive control of reentrant activity. The wave propagates upward in the tissue and downward in the simulation, mimicking a clockwise circulating reentrant wave.

Cardiac arrhythmias are disruptions in the normal initiation or propagation of electrical activity within the heart and are a major cause of mortality worldwide [1]. Among the various types of arrhythmias, those involving reentrant circuits are particularly important due to their role in life-threatening conditions such as ventricular tachycardia and fibrillation. Reentry occurs when an electrical wavefront continuously circulates within cardiac tissue (Fig 1A), either around a fixed anatomical obstacle (*anatomical reentry*) (Fig 1B) or a functional pivot point, giving rise to spiral wave patterns (*functional reentry*) [2]. These spiral waves have been shown to exist in mammalian hearts [3], including human hearts [4], which can become unstable and multiply within the myocardium, eventually leading to fibrillation: a disorganized, unsynchronized state of excitation that impairs effective cardiac contraction and may result in sudden cardiac arrest [5,6].

Understanding the mechanisms by which reentrant waves form, stabilize, and transition into fibrillation is critical for advancing both diagnostic and therapeutic strategies. However, studying these dynamics experimentally is challenging due to the complexity and variability of cardiac tissue behavior. Closed-feedback loop systems (Fig 1D), also known as coupled cardiac systems, offer a powerful framework to address this challenge by coupling living cardiac tissue with computational simulations in real-time as it has been done with live neurons [7]. These systems allow researchers to impose dynamic and physiologically relevant perturbations based on feedback from the tissue itself, enabling precise control over reentrant circuits. By manipulating features such as circuit length, conduction velocity, and refractory period in a controlled manner, coupled systems provide a unique opportunity to dissect the conditions that promote or suppress reentrant activity, and in particular, the transition to fibrillation.

A classic example of such a coupled system is the dynamic clamp, in which a computational model injects current into an excitable cell in response to its membrane voltage [8]. This approach has been successfully used in both neuronal [9] and cardiac [10] research. In cardiac electrophysiology, closed-loop coupled systems have been developed to emulate and control reentrant circuits by modifying conduction parameters during cardiac activity [11]. Early implementations used analog and digital circuitry to achieve real-time responsiveness [12,13], which evolved into software-based systems using real-time operating platforms such as RTXI [14], which was used to control in real-time discordant alternans in live Purkinje fibers [15].

A limitation of conventional stimulation methods in these systems is their reliance on electrodes, which are spatially static [15,16] and do not require short distances to control even with advanced nonlinear control methods [17]. This constraint has led to the increasing use of optogenetics, which leverages light-sensitive ion channels expressed in genetically modified cardiac cells to achieve high-resolution, spatially flexible control of excitation patterns [18,19]. Initial applications demonstrated the feasibility of using optogenetics for precise pacing and localization of pacemaker activity [20,21]. Optical dynamic-clamp methods similarly showed real-time modulation of excitability with light-based feedback [22]. Subsequent studies applied spatial light patterns to modulate tissue behavior and terminate arrhythmias [23–25]. Further, all-optical systems combined optogenetic stimulation with simultaneous optical imaging of membrane voltage and intracellular calcium through a shared on-axis optical pathway [26]. Recent advances have demonstrated that micro-LED arrays can enable high-resolution control of cardiac monolayers through spatiotemporal optogenetic stimulation [27]. However, most of these implementations were open-loop, lacking real-time responsiveness to evolving tissue dynamics.

Recent approaches have explored fully optical closed-loop systems, where high-speed cameras are used to capture electrical activity and project stimulation patterns through digital light processors [11,28]. However, these systems often depend on fixed-delay protocols, applying stimulation after a preset delay following tissue activation. Fixed-delay strategies assume linear conduction with constant velocity and ignore the complex, nonlinear properties of real cardiac tissue and complex spatial repolarization sequences. Such protocols cannot account for variability in wavefront shape, directional differences, or refractory behavior [11], and they typically stimulate the tissue at a fixed site, limiting spatial flexibility and failing to mimic dynamic wavefront interactions (Fig 1C). In parallel, opto-electronic feedback control of membrane potential has been introduced, enabling precise modulation of single-cell action potential waveforms in isolated cells or locally within multicellular preparations, although in these implementations the feedback is obtained from a single cell within the tissue [29]. More recently, integrated platforms combining optical voltage mapping, machine learning, and optogenetics have demonstrated real-time detection and adaptive correction of arrhythmic activity through closed-loop stimulation in monolayer cardiac tissue, enabling feedback-driven control of cardiac dynamics [30].

Replacing fixed delays with real-time simulations that account for evolving tissue dynamics offers a more physiologically relevant strategy (Fig 1D). However, traditional two-dimensional (2D) cardiac models are computationally expensive and require dedicated workstations, making real-time interaction impractical with standard CPU-based simulations. To overcome this barrier, Kaboudian *et al*. [31] developed Abubu.js, a WebGL-based library that enables high-speed simulations of cardiac tissue on standard GPUs. This tool supports complex models such as Fenton-Karma [32] and O’Hara–Virág–Varró–Rudy (OVVR) [33], enabling dynamic wave propagation to be computed in real-time [34]. Crucially, it enables the generation of spatially and temporally variable reentrant wave patterns that are responsive to experimental feedback.

In this study, we introduce a fully optical, coupled cardiac system that integrates Abubu.js-based real-time cardiac simulations with optogenetically modified cardiac monolayers. By using an LED matrix and a microcontroller to stimulate cardiac cells in response to both tissue activity and simulation output, our platform enables millisecond-scale bidirectional interaction. Built from inexpensive and accessible components, this system can be readily implemented across a wide range of research settings. The platform provides a robust and flexible tool to investigate fundamental questions in cardiac electrophysiology, including how reentrant dynamics emerge, evolve, and can be disrupted. By enabling controlled manipulation of these processes, the system offers a powerful framework to study, prevent, and terminate fibrillation while addressing broader mechanistic questions in cardiac dynamics.

## II. Methods

Ethics statement

All experimental procedures involving animals were conducted in compliance with the Canadian Council on Animal Care guidelines and institutional regulations. The use of postnatal mouse cardiac tissue for the preparation of ventricular monolayers was reviewed and approved by the McGill University Animal Care Committee under protocol 2018–8044 (SOP 301–01).

### A. System overview

**Fig 2:**
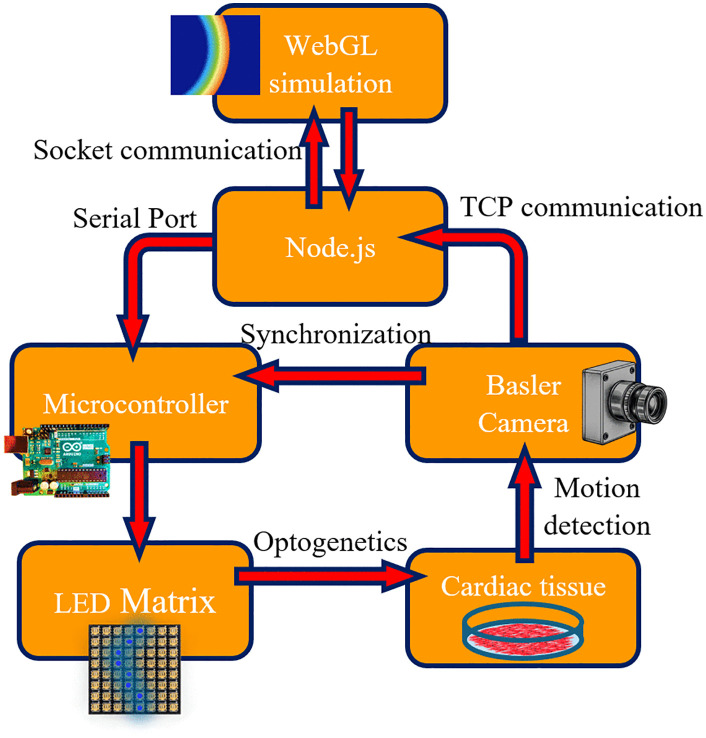
Overview of the closed-feedback loop system.

The coupled system establishes a bidirectional communication loop between the cardiac monolayer and a 2D cardiac simulation (or a fixed delay protocol), enabling real-time feedback modulation (Fig 2). A Basler camera detects tissue excitations, which are processed in parallel by a simulation and relayed to an Arduino UNO microcontroller. The Arduino triggers patterned light stimulation via LEDs onto ChR2-expressing tissue. A Node.js server coordinates data flow between components, maintaining synchronization between the camera and microcontroller for intervention within 2 ms of activity detection.

### B. Node.js communication architecture

Node.js is a back-end JavaScript runtime environment that enables server-side execution of JavaScript code outside the browser. In this system, Node.js functions as a communication hub, bridging the simulation interface with the camera, microcontroller (Arduino), and LED matrix through real-time sockets (Socket.IO), serial communication, and TCP protocols. A detailed description of the Node.js server setup and its communication architecture is available in the supplementary materials in the GitHub repository.

### C. WebGL simulation

To simulate excitation wave propagation in cardiac tissue, we used real-time 2D simulations implemented with Abubu.js, a JavaScript library built on WebGL for parallel GPU-based computation [31,34,35]. WebGL enables real-time modeling of large-scale cardiac systems by processing fragment and vertex shaders in parallel [36]. Abubu.js simplifies WebGL’s complex graphical pipeline, allowing computational variables to be stored and visualized using 4D textures. These simulations are embedded in an HTML environment and managed using JavaScript, where shaders receive and process data for each computational cell.

The server-side environment is managed using Node.js, which hosts the simulation and enables real-time communication with external hardware (e.g., microcontroller, camera) via Socket.IO and TCP protocols. The browser renders the WebGL simulation through a local server, and the main JavaScript file handles the transfer of data to shaders for visualization and control. A detailed breakdown of the Abubu.js simulation framework, folder structure, and Node.js communication architecture is available in the supplementary materials.

### D. Cardiac simulations

**Fig 3:**
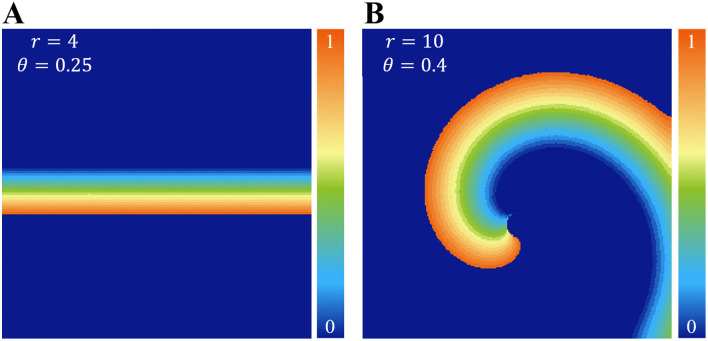
Two-dimensional cellular automata model implemented in WebGL using the Abubu.js library. **(A)** Simulation output showing the generation of uniform planar wavefronts propagating across the domain from top to bottom. The colormap represents cell voltage ranging from 0 to 1. **(B)** Simulation output illustrating the formation of spiral wave dynamics, demonstrating the model’s capability to reproduce complex reentrant patterns relevant to cardiac arrhythmias.

We implemented a cardiac model using a cellular automaton (CA) algorithm [37] within the Abubu.js library. Prior work has shown that cellular automata (CA) models can capture key features of excitable media and reproduce a range of cardiac dynamics, including burst activity, spiral waves, and fibrillation [38,39]. At the macroscopic level, these models exhibit both stable and unstable propagation patterns that are comparable to those observed in cardiac monolayers. The model simulates wave propagation in cardiac tissue, with real-time visualization enabled by GPU-accelerated WebGL rendering. Due to its simplicity, the CA approach supports rapid simulations while allowing key parameters, such as the radius of excitability (r) and the excitation threshold (θ), to be adjusted to modulate cardiac dynamics and explore different regimes of wave propagation. As a result, the model is capable of reproducing diverse physiological conditions, including unidirectional wave propagation in 2D monolayers (Fig 3A) and the generation of spiral wave dynamics (Fig 3B) similar to the experimental monolayers. The interface allows the user to create inexcitable regions using the mouse (shift-click adds a region, while control click removes a region) allowing the user to modify the shape of the simulated monolayer. In addition, the CA model can be readily replaced with alternative models described in Kaboudian et al. [34], depending on experimental needs. Further implementation details, including shader logic and model parameters, are available in the supplementary materials (Appendix A) and GitHub repository.

### E. Cardiac monolayer preparation and viral transduction

Postnatal mouse hearts (P0–P3) were isolated and enzymatically dissociated using a combination of extracellular matrix degradation and mechanical trituration to obtain a ventricular cell suspension. To minimize fibroblast contamination, cells were pre-plated on plastic for one hour before seeding. Monolayers of approximately 1 cm^2^ were cultured for recordings, with cell plating and media preparation protocols detailed in the supplementary materials, Appendix B. Cultures were maintained at 37 °C with 5% CO_2_ in a humidified incubator, and recordings were conducted in a controlled environmental chamber under the same conditions. All procedures adhered to the Canadian Council on Animal Care guidelines and were approved under McGill University protocol 2018–8044 (SOP 301–01).

Forty-eight hours after plating, ventricular monolayers were infected with adenovirus vector type 5 (dE1/D3) encoding channelrhodopsin-2 mutant H134R (ChR2(H134R)) at a multiplicity of infection (MOI) of 100. Culture media was refreshed every 48 hours post-infection. Additional details regarding infection and viral handling are provided in the supplementary materials, Appendix B.

### F. Motion detection using high-speed imaging

**Fig 4:**
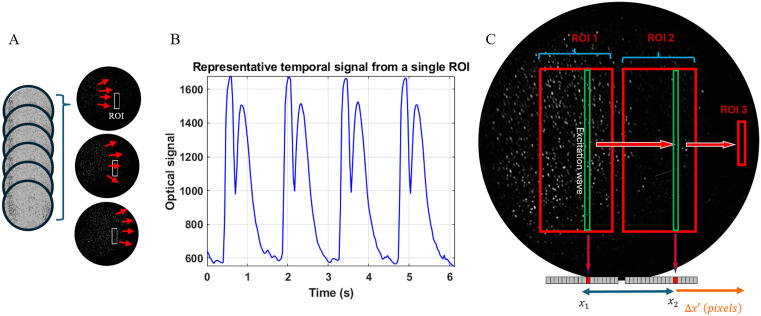
Motion-based wave detection algorithm. **(A)** Motion is extracted by subtracting the pixel intensity from its value six frames earlier, revealing tissue contractions and wave propagation. **(B)** Temporal signal from a representative region of interest (ROI, white rectangle) showing two intrinsic peaks corresponding to the contraction and relaxation phases of cardiac cells. **(C)** Excitation wave timing is determined by detecting peaks in pixel intensity across two regions of interest (ROI 1 and ROI 2), shown schematically. The algorithm calculates conduction velocity and estimates wave arrival time at ROI 3 based on spatial and temporal offsets. This value is transmitted to the feedback-loop controller.

A Basler Ace acA1920-155um camera operating at 30 frames per second (FPS) was used to capture optical signals from the cardiac monolayer. Camera configuration was performed using the Pylon 6.0.1 Camera Software Suite, with the exposure time set to 30,000 µs and digital I/O settings configured for triggering on exposure active. The system recorded 500 frames per experiment, with each frame spanning 33.33 ms. Building on previous approaches [40–42], we developed a motion-detection algorithm that identifies excitation waves from tissue displacement, rather than relying on traditional voltage-sensitive dye methods, thereby enabling cost-effective, long-duration recordings. Raw frames do not visibly reveal excitation waves; therefore, the algorithm computed the absolute difference between the current frame and its sixth predecessor (Fig 4A). This operation highlights contractile activity as regions of intensity change, effectively visualizing propagating wavefronts across the tissue.

To achieve sub-frame temporal resolution with low-cost imaging, the algorithm analyzed motion across two regions of interest (ROIs) within the tissue (Fig 4C), each measuring 700 × 300 pixels. It first detected excitation in the first ROI by comparing pixel intensity averages and identifying the pixel column where the mean surpassed a predefined threshold. The same method was then applied to the second ROI. From the spatial and temporal difference between the two detections, the conduction velocity (CV) was calculated using the formula:


$$\begin{array}{c} CV=\frac{\left(x_{\text{2}}-x_{\text{1}}\right)}{\Delta f} \end{array}$$ (1)

where $x_{\text{1}}$ and $x_{\text{2}}$ are the detected column positions and Δf is the number of frames between detections. This allowed estimation of wave arrival time at specific regions based on velocity and distance:


$$\begin{array}{c} \Delta t_{camera}=\left(\frac{\Delta x^{\prime }}{CV}\right)\times \text{3}\text{3}.\text{3}\text{3}\;ms \end{array}$$ (2)

The code and parameter settings for this algorithm are provided in the GitHub repository.

### G. Microcontroller and LED matrix

**Fig 5:**
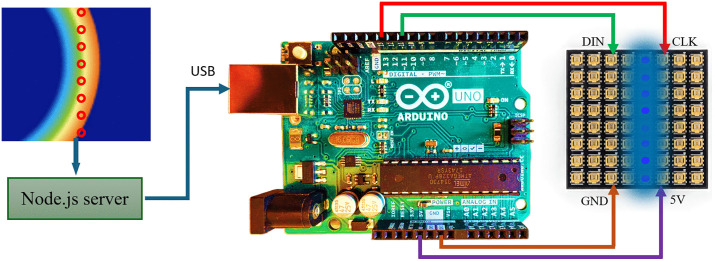
Real-time optogenetic stimulation via simulation-driven LED control. The simulation reads voltage values at specific coordinates in the 2D texture and, upon detecting activation, transmits a signal to the Node.js server. The server relays this information via serial communication to the Arduino microcontroller, which then triggers a defined pattern on the LED matrix. This enables synchronized light delivery to the cardiac monolayer based on simulated wave dynamics.

Microcontrollers are compact, fast-processing units that integrate a CPU, clock, and I/O ports to digitally control external systems, including scientific instruments, and can also be used to simulate spiral waves [43]. In this work, an Arduino UNO was programmed using Arduino IDE (v1.8.15) to control an 8 × 8 Adafruit DotStar LED matrix (shown in Fig 5). The microcontroller received buffer data via USB from the Node.js server through a serial port program. The LED matrix, soldered to the appropriate Arduino pins (GND, 5V, CLK to pin 13, and DIN to pin 11), projected blue light to activate ChR2-expressing cardiac cells. The simulation (main.js) can dynamically trigger customized LED patterns based on wave activity in real-time, enabling spatial and temporal optical control. A 3D base for the LED matrix was designed in SketchUp and printed using a Prusa MK2S printer. Implementation details and Arduino code are available in the supplementary materials on the GitHub page.

### H. Quantifying communication delays in the closed-loop hardware interface

To evaluate system accuracy, we used a photodiode (Thorlabs SM05PD1A) with a preamplifier (Thorlabs AMP 110), recorded by a digital oscilloscope (PicoScope) via the PicoLog 6 application. The photodiode captured the light output from the LED matrix with millisecond precision, registering each LED flash as a sharp drop in voltage. The intervals between successive flashes (cycle periods) were calculated and plotted in a histogram to assess timing consistency. A Python script for processing the photodiode data and generating histograms is available in the supplementary materials on the GitHub page. To identify sources of delay in the closed-loop system, we implemented three experimental designs. First, the Arduino was programmed to activate the LEDs periodically with a cycle of 1101 ms (this value was determined based on pilot experiments with the WebGL simulations) and demonstrated high temporal precision (mean cycle period = 1101.61 ms; standard deviation = 0.49 ms), confirming minimal delay in the microcontroller-LED interface. Second, periodic activation signals were sent to the Arduino via a Node.js script using serial port communication, which introduced increased variability (mean = 1105.66 ms; standard deviation = 5.62 ms), indicating latency within the communication pathway. Finally, the HTML client (running the WebGL simulation) was programmed to send periodic signals to the Node.js server, which then relayed them to the Arduino. This setup introduced additional delay and variability (mean = 1110.65 ms; standard deviation = 7.20 ms), attributed to the combined overhead of socket communication and browser execution. These findings demonstrated the need to synchronize the camera directly with the Arduino to bypass accumulated delays and preserve the temporal precision of real-time feedback.

### I. Synchronization of camera and microcontroller

To synchronize the Basler camera (acA1920-155um) with the Arduino microcontroller, a 6-wire Basler Power-I/O cable was used. The camera’s TTL output signal, generated at the start of each frame acquisition, was routed through the cable, with the brown wire connected to the Arduino’s analog input (A3) and the white wire to ground. This TTL pulse allowed the Arduino to count frames in real-time, effectively using the camera as a precise clock.

The camera software estimated the time required for a detected wave to reach a target region within the field of view ($\Delta t_{camera}$). This value was combined with the elapsed frame count (F) to calculate the total time since the beginning of acquisition: $t=\Delta t_{camera}+F\times \text{3}\text{3}.\text{3}\text{3}\;ms$. This timestamp was sent to the WebGL simulation, which modeled wave propagation across a virtual tissue domain. The simulation determined how many iterations ($F_{simulation}$) were needed for a virtual wave to travel the simulated domain. This number was scaled to milliseconds using a conversion factor dt, derived from the domain length, estimated tissue conduction velocity (CV), and number of simulation iterations ($\Delta {\text{t}}_{\text{simulation}}=F_{simulation}\times dt$).

The total time delay ($t_{total}=\Delta t_{camera}+\Delta t_{simulation}$) was then sent via serial port communication to the Arduino. The Arduino converted this delay into a target camera frame and a residual delay ($t=F_{total}\times \text{3}\text{3}.\text{3}\text{3}\;ms+Remainder$). It then waited for $F_{total}$ TTL pulses from the camera and paused for the calculated remainder before activating the LED matrix. This approach eliminated the need for immediate microcontroller responses and bypassed variable delays introduced by Socket.IO or serial transmission, thereby improving the temporal accuracy of LED stimulation.

### J. Validation of system timing using synthetic wave stimulation

To evaluate the system’s temporal precision, a DMD projector (ViALUX XGA1303) was used to generate artificial excitation waves mimicking cardiac tissue dynamics. The projector was controlled by a computer running Jython software (a Python interpreter implemented in Java), which is available for download at: https://zenodo.org/records/5800192. Periodic wave patterns with 1100 ms intervals were projected onto the microscope stage. These optical signals were recorded by the Basler camera and processed by the WebGL simulation, which was configured to return a fixed delay rather than simulate wave propagation. After this programmed delay, the LED matrix was triggered using the feedback protocol described above. Since the projected waves had constant periodicity and the system applied a fixed delay, the cycle periods detected by the photodiode were expected to remain stable. This experimental setup provided a controlled means of assessing the accuracy and consistency of the device’s response timing.

### K. Optical feedback experimental system

**Fig 6:**
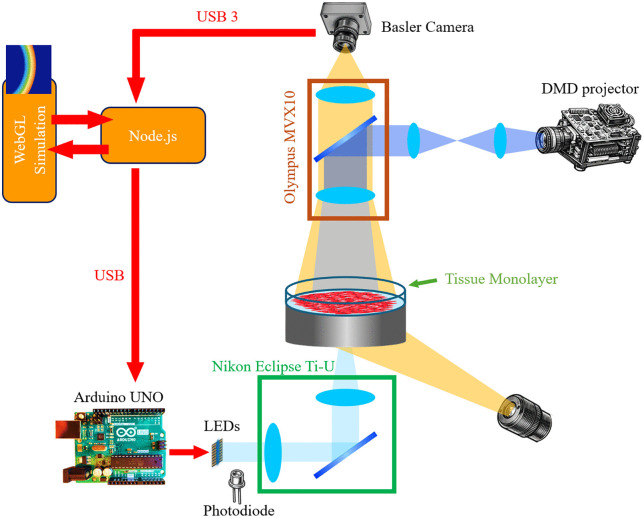
Schematic of the coupled system integrating imaging, optical stimulation, and computational simulation.

The coupled system integrates real-time optical stimulation and imaging using a fully synchronized feedback loop. As shown in Fig 6, the setup combines two microscope platforms (Olympus MVX10 and Nikon Eclipse Ti-U) to support wave imaging and optogenetic control, respectively [44]. A Basler acA1920-155um camera captures cardiac monolayer activity from above via the MVX10 microscope, while an 8 × 8 Adafruit DotStar LED matrix provides optogenetic stimulation from below through the Nikon Eclipse Ti-U’s optical path. Node.js manages bidirectional communication between the WebGL-based simulation, the camera, and the Arduino UNO microcontroller. For validation experiments, a DMD projector projects spatially controlled artificial excitation waves onto the tissue plane. A photodiode coupled with a preamplifier records LED light output to verify timing precision. All components are linked via USB or TTL signal pathways, creating a closed-loop system capable of real-time detection and feedback actuation.

## III. Results

Here, we present a closed-feedback loop system that couples a cardiac monolayer with 2D computational simulations, enabling dynamic intervention in wave propagation using a more flexible framework than fixed-delay protocols. We evaluate the accuracy of the system and provide proof-of-concept experiments demonstrating its ability to modulate cardiac electrophysiological dynamics.

### A. System timing accuracy and sources of temporal error

Precise temporal synchronization is essential for coupled cardiac systems, as even millisecond-scale deviations can alter the propagation dynamics of reentrant waves. In cultured cardiac monolayers, electrical wavefronts typically travel at CV ≈ 10 μm/ms (comparable to those in iPSC-Cardiomyocyte monolayers [45]), making synchronization critical to avoid artifacts. We evaluated system accuracy by comparing the timing consistency of projected stimulation patterns with and without camera–microcontroller synchronization (II.H). The non-synchronized system exhibited a standard deviation of 11.34 ms, calculated from the full cycle period measured using a photodiode. In contrast, synchronization reduced this deviation substantially, with a standard deviation of only 1.53 ms. This confirms that the synchronization protocol achieved the temporal resolution necessary for real-time cardiac feedback intervention.

Two principal factors influence timing precision: resolution error and signal noise. The camera’s spatial resolution (5.86 μm per pixel) limits the accuracy of wavefront position detection, with $x_{\text{1}}\approx \;\text{3}\text{5}\text{0}\;pixels$ and $x_{\text{2}}\approx \;\text{7}\text{0}\text{0}\;pixels$ (Fig 4), each having an associated uncertainty of ±5.86 μm. This corresponds to a displacement of Δx≈ 350 pixels, or approximately 2051 μm. According to the rules of propagation of uncertainties [46], the combined spatial error in wave displacement, Δx, is calculated as:


$$\begin{array}{c} \delta (\Delta x)=\sqrt{{\left(\delta x_{\text{1}}\right)}^{\text{2}}+{\left(\delta x_{\text{2}}\right)}^{\text{2}}}=\text{8}.\text{2}\text{9}\;\mu m \end{array}$$ (3)

Given a displacement $\Delta x^{\prime }\approx \text{2}\text{9}\text{3}\text{0}\;\mu m$ (Fig 4) over 6 frames ($CV=\;\frac{\Delta x}{\Delta F}$), the conduction velocity (CV) uncertainty is:


$$\begin{array}{c} \delta (CV)=|CV|\cdot \sqrt{{\left(\frac{\delta (\Delta x)}{|\Delta x|}\right)}^{\text{2}}}=\text{1}.\text{3}\text{8}\;\mu m/frame \end{array}$$ (4)

Consequently, the propagated error in timing, Δt, based on the frame rate (33.33 ms/frame), is:


$$\begin{array}{c} \delta (\Delta t)=|\Delta t|\cdot \sqrt{{\left(\frac{\delta (CV)}{|CV|}\right)}^{\text{2}}+{\left(\frac{\delta \left(\Delta x^{\prime }\right)}{|\Delta x^{\prime }|}\right)}^{\text{2}}}=\text{1}.\text{4}\text{1}\;ms \end{array}$$ (5)

**Fig 7:**
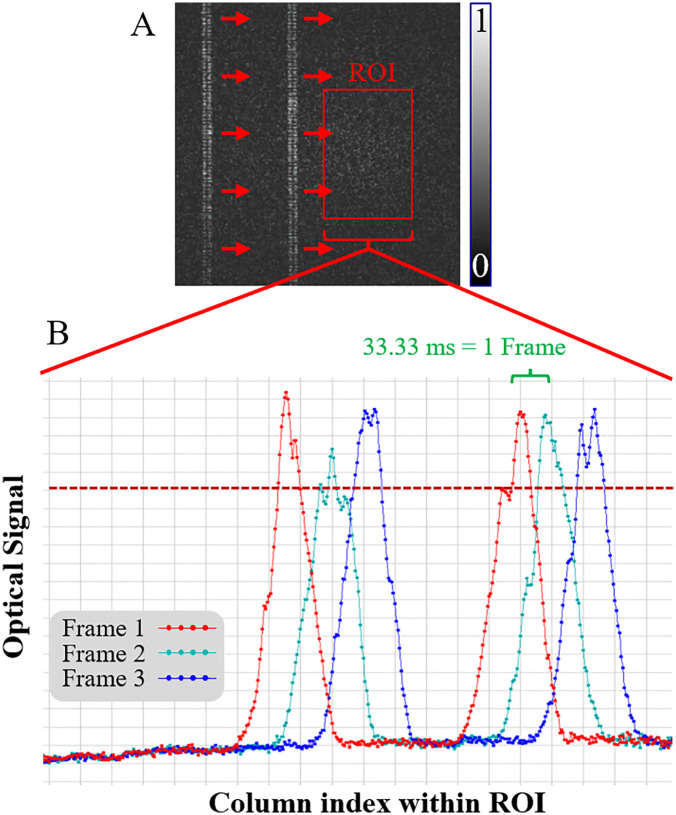
Source of inaccuracy due to signal noise. **(A)** A frame from simulated waves generated by the projector, demonstrating double peaks corresponding to the contraction and relaxation phases of virtual cardiac cells. **(B)** The plot shows the averaged optical signal across columns within the region of interest (ROI) for three consecutive frames. Due to the non-uniform shape of detected waves, increasing the detection threshold (dotted line) shifts the estimated timing of signal detection by several milliseconds, thereby introducing variability in wavefront localization and contributing to temporal noise within the system.

While this level of precision is sufficient for the experimental requirements, occasional deviations persisted even under synchronized conditions. As shown in Fig 7, signal noise in the column-averaged pixel intensities used to detect wavefronts can introduce errors in peak detection. These signals inherently contain two peaks due to contraction and relaxation phases of cardiac cells. Small fluctuations or noise-induced deflections near threshold values can alter the detected timing by several milliseconds, leading to inaccurate estimations of wave velocity.

### B. Modulating tissue dynamics using a closed-loop system with 2D cellular automata simulation

Traditional closed-loop stimulation approaches for cardiac monolayers often rely on fixed-delay protocols [11] or simplified mathematical models [47]. Here, we implemented both fixed-delay and model-based closed-loop strategies, enabling real-time bidirectional interaction between the simulation and the tissue, where stimulation is guided either by predefined timing or by model predictions.

**Fig 8:**
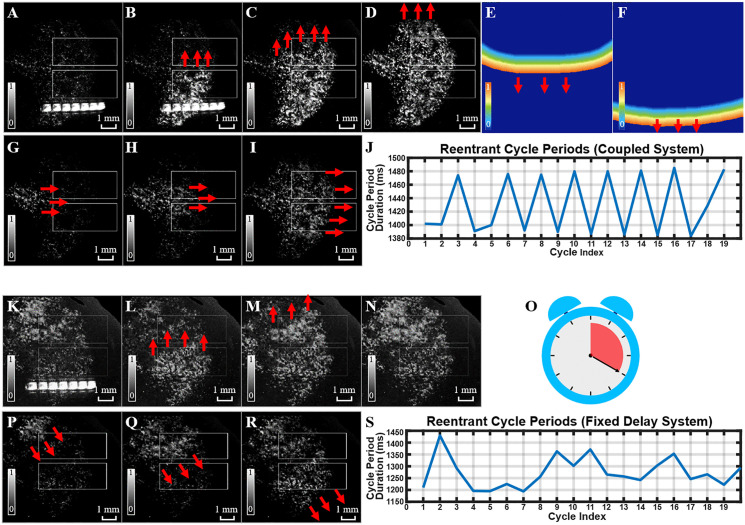
Coupled system with cellular automata model and fixed-delay protocol. **(A–F)** Snapshots of the cardiac system under closed-loop stimulation with the cellular automaton model. In **(A–D)**, two white rectangles indicate regions of interest used to estimate wave speed, assuming propagation from bottom to top; red arrows show wave direction. In **(E–F)**, simulation snapshots show waves propagating from top to bottom. **(G–I)** Tissue activity in the absence of coupling, showing wave propagation from left to right, highlighting the capability of the coupled system in modulating the tissue dynamics. **(J)** Evolution of cycle periods during model-driven feedback in **(A–F)**, exhibiting an alternating pattern. **(K–O)** Snapshots of the coupled system with fixed-delay stimulation. **(K–N)** show wave propagation in the tissue, while **(O)** illustrates the fixed delay applied by the system. **(P–R)** Tissue activity without coupling, showing wave propagation from the top left to the bottom right, representing intrinsic tissue activity in the absence of fixed-delay intervention. **(S)** Evolution of cycle periods under fixed-delay stimulation.

As shown in Fig 8A–8F, model-driven stimulation modulates wave dynamics in the cardiac tissue. Under control conditions (snapshots in Fig 8G–8I), waves propagate from left to right. Upon coupling the tissue with the simulation, the intrinsic dynamics are overridden, and wave propagation shifts to a bottom-to-top direction, as shown in snapshots in Fig 8A–8D. Corresponding simulation snapshots (WebGL Texture: 256 × 256 cells) show waves propagating from top to bottom (CA parameters: r = 4, θ = 0.25), demonstrating coordinated interaction between the model and the tissue. Together, these snapshots confirm effective coupling between the two systems. LED activation sites, indicating optogenetic stimulation, are visible in Fig 8A, 8B, and column-averaged signals were extracted from the defined regions of interest (white rectangles) in snapshots in Fig 8A–8D. The cycle length dynamics of the coupled system (Fig 8J) demonstrate alternating periods (cycle of period 2) consistent with interactions between the simulation and the biological tissue, suggestive of emergent alternans behavior in this specific experimental setting, characterized by beat-to-beat alternation in electrical activity and contraction strength at a constant heart rate [48].

In a parallel experiment using a fixed-delay protocol (100 ms delay), tissue snapshots (Fig 8K–8N) and schematic representation of the delay (Fig 8O) show that the system also modulates tissue behavior relative to control conditions (snapshots in Fig 8P–8R). In control, waves propagate from the top left to the bottom right, whereas under fixed-delay stimulation, propagation is redirected from bottom to top. However, this approach exhibits greater variability in cycle periods, as quantified in Fig 8S.

These results demonstrate that the coupled system supports real-time feedback using both model-based (Fig 8A–8F) and delay-based (Fig 8K–8O) interventions, and provide proof-of-concept evidence of its ability to intervene in and reshape tissue dynamics, highlighting its versatility for investigating complex reentrant phenomena.

## IV. Discussion

We developed a coupled cardiac system that links living monolayers with real-time 2D simulations using Abubu.js [34]. Rather than relying on predefined stimulation schemes, this platform enables controlled perturbation of cardiac wave dynamics and direct observation of tissue responses, providing a framework to investigate the mechanisms underlying wave propagation, reentry formation, and stability. This capability supports hypothesis-driven studies of arrhythmic dynamics and can be extended to more complex models, including three-dimensional (3D) representations, while maintaining real-time performance [49].

### A. Mechanistic studies enabled by the coupled system

A key advantage of this real-time interaction is that it enables computational models to directly perturb living tissue while simultaneously receiving biological feedback. Rather than treating simulations solely as predictive tools, the coupled system allows computational perturbations to influence the behavior of living tissue while simultaneously receiving biological feedback. This direct interaction bridges theoretical modeling and biological experimentation by enabling computational predictions to be evaluated immediately under identical experimental conditions, rather than through separate offline experiments.

Within this framework, physiological parameters can be modified in real-time to examine their influence on living tissue. In the cellular automata implementation presented here, parameters such as cellular excitability, refractory period, and local perturbations can be adjusted dynamically during an experiment. More physiologically detailed models, such as the OVVR model, further extend this capability by allowing manipulation of ionic conductances and other cellular electrophysiological properties. These perturbations can be introduced instantaneously through the computational model while their effects are observed directly in the biological preparation. Because these parameters can be updated continuously, the coupled system also provides a framework for investigating the effects of gradual electrophysiological changes, such as those that may occur during continuously varying drug concentrations [50] or other progressive physiological perturbations, while observing their influence on living cardiac tissue in real-time. In contrast, similar investigations have traditionally been limited either to computational simulations [51,52], where biological responses cannot be verified experimentally in real-time, or to pharmacological and biological interventions, which are often technically demanding, costly, and unsuitable for rapid, reversible parameter changes.

One particularly compelling application is the study of reentrant circuit stability, where cardiac monolayers are often too small to sustain long-lived reentry. In such cases, researchers are generally limited to *in silico* investigations [53–56], whereas the proposed framework enables these dynamics to be investigated through direct interaction between computational models and living tissue.

Finally, because both the computational model and the biological preparation preserve the spatial organization of 2D cardiac wave propagation, the platform enables investigation of spatially distributed electrophysiological phenomena. Future extensions to 3D simulations could further expand this capability by enabling investigations of how spatial organization, wavefront geometry, and tissue heterogeneity influence cardiac wave propagation and reentrant behavior. Taken together, this framework enables biological hypotheses regarding cardiac wave propagation and reentrant dynamics to be investigated through direct, real-time interaction between computational models and living tissue.

### B. Advantages over previous systems

Compared to earlier hybrid systems, the proposed setup introduces key improvements in accessibility, functionality, and integration. Prior approaches often relied on high-speed imaging equipment [11,28,47], which limits their adoption in resource-constrained environments. Our system addresses this by incorporating a custom algorithm capable of achieving high temporal accuracy using low-frame-rate machine vision cameras, thus significantly reducing experimental costs. This design achieves millisecond-level precision, with system tolerance suitable for experiments requiring fine temporal resolution at the millisecond scale. In contrast to electrode-based stimulation techniques used in earlier studies [14,57,58], our fully optical, closed-loop system responds dynamically to spatially heterogeneous tissue activity and extends beyond single-cell feedback by integrating responses over large, multicellular regions of the tissue [29]. Unlike most cardiac optogenetic platforms that apply static stimuli regardless of real-time dynamics [24,41], our design enables bidirectional interaction between the tissue and the model. The system supports both fixed-delay protocols and dynamic 2D modeling, offering a versatile platform that overcomes previously outlined constraints of fixed-delay approaches [11,28]. Further, by relying on motion detection rather than voltage-sensitive dyes, our system is capable of long-duration experiments without phototoxic effects [59]. In contrast to methods that use operator learning [60] and surrogate modeling [61], which rely on precomputed datasets for training, our coupled system directly links the tissue and the simulation in real-time without requiring prior data.

Finally, the flexibility of the system, together with the accessibility of its components, allows it to be readily reconfigured to meet different experimental needs. The implementation presented here couples a cardiac monolayer with a simulation; however, the system can be adapted so that the simulation reproduces the tissue dynamics. By tuning model parameters, the generated waves can match the shape and propagation speed observed in the tissue, enabling the development of a digital cardiac twin in future experimental implementations.

### C. Limitations of the coupled cardiac system

The coupled system presented in this study has several limitations. First, the use of motion detection in place of voltage-based imaging may introduce inaccuracies. Although this dye-free approach enables longer experimental durations, it may lack the precision of voltage recordings as the algorithm can misinterpret passive tissue motion as active wave propagation, particularly in low-contrast regions [59]. Second, the reliance on low-frame-rate cameras constrains temporal resolution. The system assumes wave concordance, i.e., constant propagation velocity across the tissue [62], which is not representative of discordant alternans, where velocity varies spatially. Also, the system assumes constant wave speed. If wave speed varies (e.g., the wave passes through a region with low conduction velocity), the prediction algorithm fails to estimate accurate wave locations, limiting the scope of applicable experiments. Third, the system is sensitive to noise: tissue samples generating significant motion artifacts can degrade detection accuracy. Although future iterations may integrate Gaussian filtering to improve signal quality, this remains an unresolved challenge. Compared to high-speed camera-based systems that achieve precise real-time wave localization [11,47], our algorithm estimates wave position using only two reference points. While this method works well for unidirectional and smoothly propagating waves, it is less effective when wavefronts change direction. Moreover, our system has only been validated using cardiac monolayers, whereas other systems have demonstrated functionality in ex vivo intact hearts [11,47]. Furthermore, the cellular automaton model cannot generate electrophysiological waves with dynamic wavelengths and conduction velocities, in contrast to differential equation-based models such as the Fenton–Karma [32] and OVVR [33] models. This limitation restricts the system’s ability to reproduce certain physiological conditions associated with impaired ion conductance in cardiac cells, which the cellular automata framework cannot accurately simulate.

### D. Future directions

This coupled system offers a powerful platform for studying the dynamics of reentrant arrhythmias and other disruptive cardiac activities. Future developments include comparing fixed-delay protocols with 2D simulations to identify the most physiologically accurate modeling approach. Enhancing stimulation precision by replicating waveforms observed in simulations may improve the stability of reentry and better mimic intact tissue behavior. Integrating AI-based computer vision for real-time detection and suppression of arrhythmic patterns [30] could further expand the system’s therapeutic potential and support the development of adaptive, feedback-driven interventions.

Beyond the cellular automata framework used in this study, the platform can be implemented with more complex, differential equation-based cardiac models, such as the Fenton–Karma [32] or the OVVR models [33]. These models offer a richer set of physiological parameters for controlling cardiac dynamics and can generate wavefronts with variable wavelength and conduction velocity, enabling closer replication of intact tissue behavior [63]. Implementing such models within the GPU-accelerated Abubu.js framework could achieve simulation speeds comparable to real tissue activity, making real-time, bidirectional coupling with living cardiac monolayers feasible [34]. In principle, this framework can also be extended to 3D cardiac models, which are computationally tractable using modern GPU resources and have been demonstrated in prior WebGL-based implementations [49]. Extending to 3D would enable more realistic representations of cardiac structure and wave propagation, offering greater insight into whole-heart dynamics and supporting more clinically relevant investigations of complex arrhythmias.

However, extending the system to a fully closed-loop 3D setting remains experimentally challenging. Optical mapping at the surface captures a depth-integrated signal, making it difficult to distinguish true three-dimensional wave behavior, and our current camera-based setup cannot access activity within the tissue volume. On the stimulation side, while surface LEDs work well for 2D preparations, targeting specific regions in depth would require more advanced approaches, such as embedded optical probes, which come with added complexity and potential damage to the tissue. For these reasons, although 3D simulations can be integrated into the framework, achieving a fully coupled 3D system will depend on further advances in volumetric imaging and stimulation methods.

## V. Conclusion

We developed a real-time coupled system that integrates living cardiac monolayers with GPU-accelerated 2D simulations to enable dynamic, closed-loop modulation of reentrant wave activity. This system introduces a novel framework that replaces fixed-delay protocols with spatially extended models capable of predicting wave propagation and interacting with biological tissue in real-time. Using a low-cost, accessible setup and custom algorithms, the system achieves millisecond-level precision without requiring high-speed imaging hardware, making it feasible for a broad range of research environments. It successfully demonstrated the generation and intervention in reentrant loops and holds potential for modeling pathological conduction conditions. By enabling bidirectional interaction between simulated and biological domains, the platform offers a versatile tool for studying arrhythmic behaviors, identifying the mechanisms that sustain them, and evaluating dynamic intervention strategies. The dye-free optical configuration supports long-duration experiments, providing opportunities for chronic studies and therapeutic development. Looking ahead, the coupled system can be expanded with more detailed simulations, 3D modeling, and real-time AI-based detection to enhance its clinical applicability. Ultimately, this work lays the foundation for adaptive, patient-specific interventions by coupling computational modeling with live cardiac tissue in a responsive and scalable framework.

## Supporting information

S1 Appendix A. Cellular automaton model implementation and code access: Detailed description of the cellular automaton (CA) model, including its theoretical framework, implementation, and parameters. The full code for all components of the coupled system is available on GitHub (https://github.com/younesvalibeigi/Coupled-Cardiac-System), along with documentation. B. Monolayer tissue preparation: Detailed procedures for neonatal cardiomyocyte isolation, tissue culture, and adenoviral infection used to generate cardiac monolayers. (DOCX)
