# Supplementary material for: Real-time GPU-accelerated coupled cardiac system: Integrating bidirectional interactions between living optogenetic monolayers and computational simulations
Source: PLoS Comput Biol. 2026 Aug 3;22(8):e1014590. doi: 10.1371/journal.pcbi.1014590 (PMC13450847; doi:10.1371/journal.pcbi.1014590)
Supplement: Supplementary file 1 [file pcbi.1014590.s001.docx]

# Appendix A. Implementation of Cardiac Models in Abubu.js

## Overview

This document provides detailed implementation information for the cardiac simulation model used in the coupled system: the Cellular Automata (CA) model. The model is implemented using the Abubu.js library for GPU-accelerated real-time simulation via WebGL shaders.

## A.1. Cellular Automaton (CA) Model

### A.1.1 Theoretical Background

Cellular automata (CA) are discrete computational models that simulate the propagation of excitation waves using simplified rules based on cell states and neighborhood interactions. CA models are computationally efficient and particularly suited for modeling large-scale tissue dynamics with minimal computational cost.

In a cardiac CA model, each cell exists in one of three basic states:

- **Excitable** (voltage < 0.05)
- **Active** (voltage = 0.98)
- **Refractory** (0.05 ≤ voltage < 0.98)

Cells are activated based on a threshold ratio of active neighboring cells within a defined radius. After activation, a refractory period prevents immediate reactivation.

### A.1.2 Model Parameters

- **Excitability radius (**$\boldsymbol{r}$**)**: Number of grid units to search around each cell (e.g., 1–10 units)
- **Threshold (**$\theta$**)**: Ratio of active neighbors required for activation (e.g., 0.1–0.4)
- **Refractory decrement**: Voltage decreases by 0.051 per time step

### A.1.3 Simulation Initialization

All cells are initialized with zero voltage. An external trigger or user click initiates excitation in a region of the grid.

### A.1.4 Shader Logic

At each iteration, every cell evaluates the number of active neighboring cells (defined as those with 0.70 < voltage < 0.99) within a specified radius (Fig S1A). If the proportion of active cells within this radius exceeds a predefined threshold, the cell transitions to the active state (voltage = 0.98) in the subsequent iteration. Cells with voltage values greater than 0.05 and less than 0.98 are considered to be in the recovery phase; these cells are refractory and cannot be reactivated. Their voltage decreases incrementally at each time step by a fixed value (ΔV = 0.051) until they return to the resting state. The fragment shader (GLSL) implements the following logic for each cell:

if (voltage < 0.05) { // Cell is excitable

if (active_neighbor_ratio > threshold) {

voltage = 0.98; // Activate cell

}

} else {

voltage -= 0.051; // Recovery phase

}

Each simulation step involves two render passes to alternate between textures and allow iterative updating. The full implementation is available at: <https://github.com/younesvalibeigi/Coupled-Cardiac-System>. Fig S1 illustrates how the implemented algorithm enables wave propagation across the tissue.

**A**

Fig 1. Cellular automata model. (A) At each iteration, every cell evaluates the number of active neighbors within a defined radius R. If the proportion of active cells exceeds a specified threshold, the cell transitions to the active state in the next iteration. (B) Visualization of two consecutive iterations, where a cluster of active cells (red) triggers activation in the surrounding region, leaving cells in the refractory period behind (blue). (C) Depiction of the model’s state transitions: once a cell is activated, its voltage rises to 0.98 and then progressively declines with each subsequent iteration, representing the refractory phase.

Refractory state

**B**

**C**

Excitable state

### A.1.5 Performance

The CA model runs at real-time speeds on standard GPUs (e.g., 60 FPS on NVIDIA GTX 1050) and is suitable for high-resolution grids (e.g., 256×256). This computational efficiency arises from the simplicity of the CA formulation, which enables parallel implementation on GPU hardware with minimal resource requirements.

The parameters of the model are fully adjustable and can be tuned to approximate relevant dynamical properties of cardiac tissue. In the CA framework, the conduction velocity (CV) for a smooth wavefront can be approximated as proportional to $(r\cdot\frac{1-\theta}{1+\theta})$, where $r$ is the excitation radius and $\theta$ is the excitation threshold. In our implementation, using $r=4$ and $\theta=0.25$, this yields a propagation of approximately 2.4 cells per iteration. With a temporal discretization of 4 ms per iteration, this corresponds to 0.6 cells/ms. Assuming a nominal spatial scale of 20 µm per cell, the effective propagation speed is approximately 12 µm/ms (12 mm/s). These values can be rescaled by adjusting either the spatial mapping or the temporal discretization.

Similarly, the action potential duration (APD) is determined by the recovery dynamics of the CA model. With an active state of 0.98, an excitable threshold of 0.05, and a recovery decrement of 0.051 per iteration, the system requires 19 iterations to return to an excitable state. Given a time step of 4 ms per iteration, this corresponds to an APD of approximately 76 ms.

It is important to note that in this work, the model parameters were not tuned to quantitatively match the specific electrophysiological properties of the experimental monolayer (e.g., conduction velocity, restitution, or geometry). Instead, they were selected to produce stable and observable excitable dynamics within the constraints of real-time interaction. The framework is designed to remain flexible, allowing users to adjust parameters to better reflect specific experimental conditions when required.

## A.2. Access to Code

The complete source code for the model, including GLSL shaders, main.js, and configuration files, is available at:

<https://github.com/younesvalibeigi/Coupled-Cardiac-System>

Implementation examples and documentation are also provided to assist new users in customizing the simulation for their own research.

# Appendix B. Monolayer preparation

The following protocol follows a step-by-step procedure from Miltenyi Biotec (Protocol No. 130-098-373, Miltenyi Biotec Inc., California, US), with additional steps from Ambrosi et al. (2014) and Burton et al. (2015). The entire protocol was first described in a submitted MSc thesis from a student in the same laboratory (Sepúlveda, 2020).

Cells were isolated using a Miltenyi gentleMACS Dissociator, which automates the cell dissociation process. Procedures for animal handling were performed in agreement with guidelines of the Canadian Council on Animal Care. Neonates were euthanized by decapitation in agreement with McGill University SOP 301-01 under approved protocol 2018-8044. Sterile techniques should be followed during all procedures.

## B.1. Tissue culture coating (for 24-well plates) and plating cell densities:

1. 5 μL of fibronectin (VWR, Ontario, CA) is diluted into 500 μL of phosphate buffered saline (PBS) for a final concentration of 50 μg/μL.
2. 125 μL of the thoroughly mixed dilution must be added to each well.
3. The plate should be incubated at 37 ºC and 5% CO2 for at least 1 h before use.

The densities described in the table below were used for neonatal mouse cardiomyocytes.

| Cell density and seeding volume (Table B1) | | | |
| --- | --- | --- | --- |
| Vessel size | Surface area in cm^2^ | Seeding volume in ml | Cell number (~$156\times{10}^{3}$ cells/cm^2^) |
| Glass ring | 1 | 0.232 | $156\times{10}^{3}$ |
| 24-well | 1.9 | 0.6 | $296\times{10}^{3}$ |
| 12-well | 3.8 | 1.2 | $593\times{10}^{3}$ |

## B.2. Ventricle dissociation, tissue culture, and adenoviral infection of cardiac monolayers:

i. Prepare necessary solutions from stocks: Penicillin/Streptomycin Mixture (Pen/Strep, Quality Biological, Cat#: 120-095-721), Dulbecco's Modified Eagle Medium (DMEM, Wisent, REF: 319-062-CL), Phosphate Buffered Saline (PBS, Wisent, REF: 311-010-CL), and Fetal Bovine Serum (FBS, Gibco, Cat#12483020). DMEM (10%) maintenance media is prepared by adding 50 mL of FBS and 5 mL on Pen/Strep to 450 mL of DMEM, and DMEM (2%) is prepared by adding 200 μL of FBS and 100 μL of Pen/Strep to 10 mL of DMEM.
ii. Harvest the heart and dissect the ventricles:
    a. Obtain a litter (6 or more animals) of postnatal 0 – 3 (P0 - P3) day old mice pups.
    b. Pups are decapitated, and the heart is isolated by first opening the rib cage with sharp scissors and removing the heart with tweezers. The hearts are placed in a shallow plate containing 3 mL of PBS solution on ice.
    c. Scissors are used to remove the ventricles (around the lower 70% portion of the heart) and remaining connective tissue.
    d. The tissue is cleaned by swirling the plate regularly to remove blood cells from the ventricles.
    e. The ventricles are each cut into 4 – 6 small pieces around 1–2 mm³.
iii. The ventricles are dissociated into single cells using the Neonatal Heart Dissociation Kit and gentleMACS Dissociator, following protocol 130-098-373. The program used on the dissociator was “m_neoheart_01_01”. The protocol takes approximately 45 minutes.
     a. The resulting suspension is centrifuged at $600\times g$ for 5 min.
     b. The pellet is resuspended in 10 mL of DMEM 10% using gentle manual agitation with a wide-mouthed pipette.
iv. Remove cardiac fibroblasts and count cells:
    a. The 10 mL suspension is transferred to a shallow 10 cm culture dish and incubated at 37 degrees and 5% CO_2_ for 45 minutes. Fibroblasts settle and adhere to the bottom of the dish. The supernatant contains an enriched population of myocytes.
    b. The supernatant (10 mL) and an additional 5 mL PBS for washing the plate are transferred to a 50 mL Falcon tube.
    c. Centrifuge the 15 mL supernatant at $600\times g$ for 5 min and resuspend the pellet in 1 mL of warmed DMEM 10%.
    d. Cell concentration is determined by using a standard hemocytometer, with Trypan Blue added to count dead cells.
v. Culture cardiomyocyte cells in 24-well plates:
   a. Once counted, the desired concentration per well is determined by table B1 above (approximately 300,000 cells per well in a 24-well plate).
   b. An additional 1 mL of DMEM 10% per well is added, and the tissue is incubated for 24 hours.
   c. Provide post-plating maintenance by replacing the solution with 2 mL of DMEM 10% every 24-48 hours.
vi. Infect the ventricular myocytes with Ad-CMV-hChR2(H134R)-eYFP (Ambrosi et al., 2014) 2 to 3 days post plating:
a. Replace DMEM 10% with a small amount of DMEM 2% (250 μL per plate).
b. For our desired multiplicity of infection (MOI) of 100 and a virus titer of$2.2\times{10}^{6}$ virus/μL, we use 13.6 μL of virus solution per well (assuming 300,000 cells/well).
c. Incubate monolayers for 48 h prior to running experiments

## B.3. External References

1. Ambrosi, C.M. and E. Entcheva, *Optogenetic Control of Cardiomyocytes via Viral Delivery.* Methods in molecular biology (Clifton, N.J.), 2014. **1181**: p. 215-228.
2. Burton, R.A.B., et al., *Optical control of excitation waves in cardiac tissue.* Nature Photonics, 2015. **9**(12): p. 813-816.
3. Sepúlveda, J.R., *Optically induced heterogeneities in cardiac tissue*. 2020, McGill University: Montreal. p. 76.
