# Supplementary material for: Real-time GPU-accelerated coupled cardiac system: Integrating bidirectional interactions between living optogenetic monolayers and computational simulations
Source: PLoS Comput Biol. 2026 Aug 3;22(8):e1014590. doi: 10.1371/journal.pcbi.1014590 (PMC13450847; doi:10.1371/journal.pcbi.1014590)
Supplement: Supplementary file 3 [file pcbi.1014590.s004.docx]

# Responses to the Second Round of Reviews:

**Reviewer #1:**

**My concerns have broadly been addressed through clarification of the paper’s claims.
However, I would still have liked to see either an experiment that would have been difficult to perform without the simulator, or a clearer demonstration that the system provides new biological insight.**

To address this concern, we have added a new subsection, "Mechanistic Studies Enabled by the Cardiac Coupled System," to the Discussion.

In this new section, we discuss the study of re-entrant circuit stability, where conventional cardiac monolayers are often too small to sustain long-lived re-entry, leaving researchers largely limited to *in silico* investigations. We also highlight how the proposed framework enables continuous modulation of electrophysiological model parameters, allowing gradual physiological changes, such as those associated with continuously varying drug concentrations, to be applied while simultaneously observing their effects on living tissue in real time. Such studies are difficult to achieve using biological preparations alone, where rapid and reversible manipulation of physiological parameters is often impractical, or with previous closed-loop systems based on fixed-delay stimulation, which do not permit continuous modification of the underlying electrophysiological properties during an experiment. Finally, we describe how the computational model provides spatial control over cardiac wave dynamics, enabling investigations of how spatial wave patterns, wavefront geometry, and tissue heterogeneity influence cardiac activity through direct interaction with living tissue. We believe these additions more clearly demonstrate the biological significance of the proposed framework and the unique mechanistic investigations it enables.

# Responses to the First Round of Reviews:

**Reviewer #1:**

We sincerely appreciate the reviewer’s thoughtful and constructive feedback. As reviewers 1–3 consistently highlighted concerns regarding our use of the term “digital twin,” we carefully reconsidered how we describe the system. While our platform is capable of replicating aspects of tissue dynamics in real time within the simulation, its primary function is to establish a bidirectional interaction between the computational model and the cardiac monolayer. For this reason, we believe that describing it as a coupled cardiac system more accurately reflects its design and purpose.

Our goal was to develop a system that enables this form of coupling using inexpensive and accessible components, making it feasible for a broader range of researchers and laboratory settings. We demonstrate that the system can effectively influence and modulate monolayer dynamics in real time. Future work can build on this framework to address specific experimental questions through targeted simulation dynamics and controlled tissue interventions, while progressively improving fidelity between the simulation and biological tissue, ultimately moving toward a digital twin representation when appropriate.

**The paper descries a highly novel and innovative experimental – simulation hybrid system that would allow for sophisticated experiments. I have some general concerns that would be good to address.**

**1. The authors make a number of statements which may be true, but are not supported by evidence. For example, they could have created a digital twin of the simulation system to explore how delays, resolutions, or other differences impact the results.**

We are grateful to the reviewer for raising this important point. Our primary objective in this study was to develop and demonstrate a technology that can be implemented using inexpensive and accessible materials, and to show that the system functions reliably in a simplified setup as a proof of concept. The focus of this work was therefore on establishing the feasibility and usability of the platform, rather than systematically evaluating the effects of parameters such as delays, resolution, or model fidelity on cardiac dynamics. We agree that exploring how these factors influence system behavior would be valuable. However, we consider this an important direction for future work building on the present framework. To address this concern, we have revised the Introduction and Discussion to more clearly state the scope and objectives of this study, and to avoid potential misunderstandings regarding the intended role of the system.

**2. At present, they claim the system needs to be fast and then make it faster. This means we are told how accurate the system is, but no quantitative metric or formal evaluation is defined to demonstrate that it is fast enough.**

We acknowledge that this may not have been clear in the original manuscript. In the Results (Section A), we provide a detailed evaluation of system accuracy and timing error. Specifically, we generated artificial waves with fixed periods using a projector and quantified the latency and variability of the optical stimulation pipeline across the full closed-loop system, both with and without synchronization between the camera and microcontroller.

In the unsynchronized configuration, we observed a temporal jitter with a standard deviation of 11.34 ms over one full cycle of wave propagation through both the tissue (simulated using projected waves) and the computational model. After introducing synchronization between image acquisition and microcontroller triggering (with the projector substituting for the tissue in this validation), the temporal variability was substantially reduced to 1.53 ms. In addition, direct error measurements reported in Section A through equation 3-5 show a total system timing error of 1.41 ms (a typographical error in the calculation has been corrected), indicating millisecond-level precision.

We believe that the discrepancy between the measured variability and the calculated error arises from the non-uniform shape of the detected wavefront (Fig. 7). Overall, these results demonstrate that the feedback loop operates within the temporal resolution required for cardiac electrophysiology experiments.

**3. The paper describes a model–experimental coupling, but it does not describe a digital twin. A digital twin should represent the experiment, not act as an additional pathway within it. The authors should rename and rephrase this element of the paper to avoid confusion.**

In response to this and other reviewer’s feedback, we have revised the manuscript to describe our system as a coupled cardiac system, not a digital heart twin. This more accurately reflects its current design and function. We have also expanded the Discussion to better highlight the flexibility of the platform. The system can be readily coupled with different types of computational models and adapted to a range of experimental applications. In addition, it provides a foundation that can be easily extended in future work toward a digital twin.

**4. The results section does not show how well the digital twin replicates the experimental set-up. This critical result is therefore absent from the paper.**

As noted above, we have revised the manuscript to describe our system as a coupled cardiac system rather than a digital twin. As such, the goal of this study is not to achieve one-to-one replication of the experimental tissue within the simulation. Instead, our objective is to demonstrate that a bidirectionally coupled system can be implemented using inexpensive and accessible components, and that it functions reliably to interact with and influence tissue dynamics in real time. The Results section is therefore focused on validating system functionality and responsiveness, rather than replication fidelity. Future studies can build on this framework to design experiments tailored to specific questions. We have revised the Introduction and Discussion to clarify the scope of the work and to prevent further misunderstanding. We thank the reviewer for raising this source of misunderstanding.

**5. The authors do not define target accuracies or tolerances for the twin to be effective, making it impossible to statistically evaluate the twin.**

In response to this and other valid concerns we refocused the study on characterizing the temporal precision of the coupled system. As reported in Results (Section A), the system achieves millisecond-level accuracy using inexpensive components and relatively low camera frame rates. Importantly, the performance of the system depends primarily on the timing of the final tissue update via optogenetic stimulation at the end of the loop. While intermediate computations, such as wave detection and simulation updates, can occur in advance, accurate control is maintained as long as the timing of the optogenetic stimulation is preserved. To support this, we implemented a prediction algorithm that estimates wave speed and location, allowing the simulation to update ahead of time. Combined with the fast execution of the GPU-based model, this ensures that stimulation is delivered at the correct moment despite hardware constraints. As a result, the system demonstrates millisecond-level precision and provides sufficient tolerance for experiments requiring this temporal resolution. We have clarified this point in the revised manuscript to better define the operational accuracy and its relevance to the intended applications.

**6. No biological insight is presented. The authors may want to consider whether this work is more suitable for an IEEE journal rather than a computational biology journal.**

We appreciate the reviewers concerns regarding the relevance of our work for this journal. While the present study focuses on the development and validation of the system, its primary purpose is to enable the investigation of biological questions in cardiac electrophysiology. The coupled framework allows direct interaction between a living cardiac monolayer and a computational model, creating an experimental setting where hypotheses about wave propagation, reentry formation, and intervention strategies can be tested in real time. In this sense, the computational component is not used solely as an engineering tool, but as a biological modeling framework that interacts with experimental tissue to probe underlying dynamics. This type of integration, linking in vitro systems with real-time computational models, is closely aligned with the goals of computational biology, where models are used to understand and interrogate biological systems. We agree that the current manuscript emphasizes the methodology, and to make this clearer, we have revised the Introduction and Discussion to better highlight the biological questions that this platform is designed to address, as well as its potential for studying cardiac dynamics and control mechanisms.

**7. Finally, the digital twin was not tuned to represent the experiment. If this tuning was performed, substantially more detail should be provided.**

As clarified in our revisions, the system was not designed to be tuned as a high-fidelity replica of the experimental tissue. Instead, our goal was to establish a bidirectionally coupled framework in which the computational model interacts with and extends the behavior of the cardiac monolayer, rather than reproducing it. In this context, the simulation serves as an active component of the system, enabling controlled perturbations and dynamic interactions with the tissue. For the objectives of this study, we focused on demonstrating that this coupled system is functional and can reliably influence tissue dynamics in real time. We have revised the manuscript to make this distinction clearer and to avoid suggesting that a fully tuned digital twin was implemented. However, this framework can be further developed in future studies to incorporate higher-fidelity calibration when replication of tissue dynamics is required for specific applications.

**Reviewer #2:**

**In the manuscript “Real-Time GPU-Accelerated Digital Heart Twin: Integrating Bidirectional Interactions Between Living Optogenetic Monolayers and Computational Simulations” by Younes Valibeigi and co-workers addresses a relevant research topic of potentially broad interest.

 A digital twin is a dynamic, virtual replica of a physical system that is continuously updated with real-time data from its physical counterpart. Sensor Data from the physical system is fed into the digital twin in real-time to reflect the current state.
 In the system investigated in this manuscript, the physical system is a two-dimensional cardiac optogenetic cell culture. The state of the system is obtained by optical sensors (digital camera), that measure the mechanical deformation of excitable cells. Actuators are multiple LEDs in an array that can activate optogenetically modified channels. The digital twin is implemented for simplicity as a cellular automaton.

 While the research question is highly relevant, I have some concerns regarding the methods and results. I suggest a major revision of the manuscript.**

Reviewer 2 raised several important issues that made us rethink how our research is presented, in addition to restating concerns raised by reviewer 1. This revised submission reframes the work as a coupled cardiac system and clarifies several key points raised by the reviewer. We sincerely appreciate the reviewer’s thoughtful and constructive feedback, which have led to an improved submission.

**1. Additionally, several variants of the digital twin methodology have already been published, most recently for example** [**https://lnkd.in/e9GYsTmW**](https://lnkd.in/e9GYsTmW) **Therefore, the authors should further highlight their innovation.**

We appreciate the reviewer for bringing this relevant and recent work to our attention. At the time we prepared our manuscript, this study had not yet been published, although it references our preprint. We have now incorporated this work and clarified how our approach relates to and differs from existing methodologies, in order to better highlight the specific contributions and innovations of our system.

**2. From the data presented in the manuscript, it remains unclear that a digital twin has been implemented, i.e., a virtual replica of the observed biological system.**

As reviewers 1–3 consistently highlighted concerns regarding our use of the term ‘digital twin’, we carefully reconsidered how we describe the system. While our platform is capable of replicating aspects of tissue dynamics in real time within the simulation, its primary function is to establish a bidirectional interaction between the computational model and the cardiac monolayer. For this reason, we believe that describing it as a coupled cardiac system more accurately reflects its design and purpose.

**3. To what extend does the dynamics of the numerical simulation represent the observation?**

Prior work has shown that cellular automata (CA) models can capture key features of excitable media and reproduce a range of cardiac dynamics, including burst activity, spiral waves, and fibrillation [1, 2]. At the macroscopic level, these models exhibit both stable and unstable propagation patterns that are comparable to those observed in cardiac monolayers. In our framework, the CA model is used to extend and interact with the tissue. Based on the established properties of CA models, it provides an appropriate level of abstraction to reproduce and manipulate relevant cardiac behaviors within the coupled system. We have clarified this point in the revised manuscript to avoid potential confusion.

**4. The observed quantity is the mechanical contraction of the cells. However, the model describes a generic excitable medium. How do the simulation relates to the properties of the cell culture, for example, geometry of the culture, or dynamical properties such as the propagation of electrical or mechanical waves, restitution dynamics for APD and CV etc.?**

In our experimental setup, the observed signal is the mechanical contraction of the cardiac monolayer. Prior studies have shown that contraction patterns in such preparations closely reflect the underlying electrical activity, allowing optical measurements of motion to serve as a proxy for voltage propagation [3, 4]. In parallel, cellular automata (CA) models are well-established representations of excitable media and have been shown to reproduce key aspects of cardiac wave propagation, including the spread of activation across tissue [1, 2].

That said, in the present work, our goal was not to tune the simulation to quantitatively match the specific properties of the cell culture (e.g., geometry, conduction velocity, or restitution dynamics). Instead, we aimed to demonstrate that the coupled system is functional and that the simulation can interact with the tissue in real time. For this reason, the simulation parameters were selected to provide stable and observable dynamics within our computational constraints, rather than to replicate the exact physiological values of the monolayer.

Importantly, the parameters of the CA model are fully adjustable and can be set by the user depending on the experimental question. For example, as described by Bub et al. [1], when the excitation radius is $r$ and the excitation threshold is θ, the conduction velocity (CV) in the CA model (for a smooth wavefront) can be approximated as proportional to $\frac{r.(1-\theta)}{1+\theta}$ per iteration. In our implementation (Results, Section B), we used $r=4$ and $\theta=0.25$, which yields a CV of 2.4 cells per iteration. By assigning a time step of 4 ms per iteration, this corresponds to 9.6 cells/ms. Assuming a nominal cell size on the order of 20 µm, this gives an effective propagation speed of ~192 µm/ms (192 mm/s). These values are not fixed and can be rescaled by adjusting the model parameters and time mapping.

Similarly, the action potential duration (APD) in the CA model is determined by the recovery dynamics. In our setup, with an active state of 0.98, an excitable threshold of 0.05, and a recovery step of −0.051 per iteration, the system requires 19 iterations to return to an excitable state. With a time step of 4 ms per iteration, this corresponds to an APD of 76 ms. Again, these values are adjustable and can be modified to better match specific experimental conditions if desired.

We have clarified in the revised manuscript and supplementary materials that the CA model is used here as a flexible and controllable representation of excitable dynamics, rather than a calibrated replica of the biological tissue. The strength of the system lies in this flexibility, allowing users to define parameters that are appropriate for their specific experimental goals. We added the above detailed calculation in the supplementary materials.

**5. If Fig. 3 represents possible states of the digital twin, how do these states quantitively compare to observed wave dynamics in the cell culture (for example with respect to spatial-temporal scales, wave lengths etc.) shown in Fig. 8?**

In the present study, our objective was not to quantitatively match the simulated dynamics to those observed in the cell culture, but rather to demonstrate that a parametrizable simulation can be coupled in real time with living tissue. Parameters such as excitation radius and threshold directly influence wave properties, including wavelength, propagation speed, and stability, and can be adjusted depending on the experimental goal.

Fig 3 is intended to illustrate representative behaviors of the CA model in isolation, independent of the coupled system. Specifically, it shows examples of distinct dynamical regimes, including linear wave propagation and spiral wave patterns. To make this clearer, we have revised Fig 3 to explicitly include the corresponding simulation parameters. We have also updated Figure 3B to show spiral dynamics consistent with wave patterns that generate tachycardia, in contrast to the stable, unidirectional waves shown in Figure 3A.

For Fig. 8, we now include the parameter values used in the coupled experiments in the manuscript. In the current implementation, the camera-based detection assumes a constant conduction velocity (CV) and a smooth, unidirectional wavefront. This allows us to estimate when the wave reaches the boundary of the tissue, even at relatively low camera frame rates. In contrast, the simulation parameters were not selected to reproduce a specific physiological regime, but rather to demonstrate that the system can operate and interact with the tissue using a given set of parameters. With these settings, we show that the system can modify tissue dynamics in real time, including overriding the intrinsic pacing activity as a proof of concept. In this context, the simulation is not intended to replicate the tissue, but to act as an extension of it. The parameters are chosen for flexibility rather than strict physiological matching, allowing users to adjust them and explore their effects on tissue dynamics in experiments tailored to specific research questions. We have clarified these points in the revised manuscript to better distinguish between illustrative simulation behavior and the goals of the coupled experiments.

**6. The dynamics of cell cultures depend on several parameters, such as time of cultivation, cell density, homogeneity etc. and are expected to vary from experiment to experiment. How are these parameters reflected in the model and how are parameters (such as diffusion constant/tensor) estimated from the observation? Again, a direct comparison of model state and observations (e.g. time series of model output and observation) could be helpful to answer the question.**

The current submission reframes the experiment as a coupled system rather than a digital twin. The simulation is therefore not intended to replicate the tissue dynamics (as it would with a digital twin), but to act as an extension that interacts with the tissue to complete and manipulate reentrant activity. In this framework, simulation parameters are not directly estimated from experimental measurements, but can be adjusted by the user to generate specific dynamics in the tissue. In the CA model, there is no explicit diffusion constant; instead, excitability and propagation are controlled through parameters such as the radius of activation ($r$) and the activation threshold (θ). By tuning these parameters, users can modify wave speed, stability, and overall behavior, either to approximate the tissue dynamics or to explore alternative regimes.

If desired, a closer match between the simulation and the tissue can be achieved by aligning measurable quantities such as conduction velocity (CV). For example, CV estimated from the tissue using the camera can be related to the model through the known relationship ∼ $\frac{r.(1-\theta)}{1+\theta}$ per iteration, allowing users to adjust parameters accordingly. Finally, for studies requiring more biophysically detailed models (e.g., including diffusion terms), we provide code and instructions in our GitHub repository to integrate other models implemented in Abubu.js, such as those described in Kaboudian et al. [5]. This enables users to extend the framework to more complex and physiologically detailed simulations when needed.

**7. Furthermore, to what extend can the model predict the dynamics of the system? More specifically, Fig. 8B shows observed waves traveling upward in the cell culture image. The simulation shows a wave (with scale bar missing) apparently showing wave propagation in the simulation. The simulation is shown a wave (colorbar missing). Is this simulation snapshot supposed to show a replica of the observed state? If so, how?**

We thank the reviewer for this helpful point regarding potential difficulties in interpretating Fig. 8 and the role of the simulation in relation to the observed tissue dynamics. To address this, we have updated Fig. 8 to include the appropriate scale and color bars, which were missing in the original version. As clarified in the revised manuscript, the simulation shown in Fig. 8 is not intended to replicate the observed state of the tissue. Instead, it serves as an extension of the tissue within a bidirectionally coupled loop. In the proof of concept experiment, optical stimulation from the LED array initiates a wave in the tissue, where the LED lights can be seen in Fig 8, camera frame. The generated wave then propagates upward and is detected by the camera. The algorithm then predicts when the wave will reach the upper boundary. In parallel, the simulation generates a corresponding wave at the simulation’s top boundary that propagates downward, forming the complementary half of a reentrant loop. Because the simulation runs efficiently on WebGL, it can complete this computation rapidly, allowing the system to maintain real-time interaction.

Once the simulated wave completes its trajectory, the system combines the estimated propagation time in the tissue (from camera prediction) and the time from the computational simulation to determine the correct timing for the next optogenetic stimulation. This timing is sent to the Arduino controlling the LED array, which delivers stimulation at the appropriate moment to close the loop and reinitiate activity in the tissue.

In this framework, the simulation is not used to predict the exact state of the tissue, but to extend its dynamics and enable controlled feedback. The snapshot shown in Fig. 8 represents the state of the simulation during this process and is intended as a schematic illustration of the coupled interaction, rather than a one-to-one match with the experimental image. The timing and spatial patterns in the simulation are therefore not expected to be identical to those in the tissue. We have revised both Fig. 8 and the corresponding description in the manuscript to make this distinction clearer and to avoid further confusion.

**8. Also, additional data that goes beyond an anecdotal example should be provided.**

As noted above, we’ve clarified that the goal of this study is to introduce and demonstrate a coupled system that enables a closed feedback loop between a cardiac monolayer and a computational simulation. In this framework, the simulation extends the tissue dynamics to form a reentrant loop, while allowing the user to control parameters within the computational domain.

Our intention in Fig. 8 was to provide a clear proof of concept showing that the system is functional, specifically, that it can interact with and modulate tissue dynamics in real time, including overriding the intrinsic pacing of the monolayer. Given this objective, we focused on demonstrating feasibility and robustness of the approach rather than performing repeated trials of the same experimental condition.

We agree that systematic exploration of different parameter regimes and conditions would be valuable. However, we believe that such studies are best suited for future work building on this platform, where specific biological questions can be addressed using tailored experimental designs. The current manuscript is intended to establish the method and its capabilities, and to show that it can be implemented using accessible and inexpensive components. To make this scope clearer, we have revised the manuscript to better distinguish between the proof-of-concept validation presented here and the broader range of experiments that this system enables.

**9. Furthermore, it is not clear that the Fig. 8 confirms that the digital twin can support model-based control, as claimed by the authors. The authors should elaborate how data shown in this figure demonstrate successful control? If control is shown, which is the desired target dynamics?**

We have reframed the system as a coupled cardiac system rather than a digital heart twin in response to this type of valid concern. We have clarified that this study’s aim is not to demonstrate model-based control in the strict sense, but to establish a functional closed-loop framework in which the simulation extends the tissue to form a reentrant circuit.

Figure 8 is intended to show that this closed-loop interaction is operational. In this setup, the system generates a circulating wave that propagates through both the tissue and the simulation, effectively mimicking a reentrant pathway. Importantly, the system is able to override the intrinsic pacing activity of the monolayer: while spontaneous activity initially originates in the upper left region of the tissue, the coupled system reinitiates activation from the lower region, demonstrating that the feedback loop can influence and redirect tissue dynamics. For the specific parameters used in this proof-of-concept experiment, we also observe patterns consistent with alternating wave behavior when analyzing the reentrant cycle. However, we emphasize that these dynamics are not presented as a targeted control objective, but rather as an example of how the system can modulate tissue behavior. Our primary objective here is to demonstrate feasibility, that the system can establish and sustain a closed-loop interaction capable of influencing tissue dynamics. The framework is designed so that the user can define the simulation parameters, and therefore the desired dynamical regime, in future studies. This enables future studies to vary simulation parameters and investigate which dynamical regimes can produce desired control states. We have revised the manuscript to clarify these points and to avoid suggesting that model-based control, in the strict sense, is demonstrated in this work.

**11. The SNR of these waves appears to be rather low in the observed wave patterns shown in Fig. 8. Low SNR requires to average wave dynamics over large regions of interests. Without spatial scales provided (wave length of excitation wave, size of roi etc.), it remains unclear what the limitations are. In this regard, the conduction velocity measurement assumes planar waves and estimates only one velocity component. The waves in the experiments, however, appear not be planar (see Fig. 8). If remains unclear how, given these significant limitations, spatial-temporal control of complex patterns can be achieved.**

We agree that variations in wave shape and velocity impose limitations on our method, and these were not made clear in our original submission. Our simplified experimental approach is motivated by our aim to use inexpensive video cameras and other off-the-shelf equipment for this study. This required us to assume a planar wave and estimate a single component of conduction velocity (CV) using two regions of interest (ROIs), as shown in Fig. 4. By measuring the time difference between these ROIs, we obtain an estimate of propagation speed under this assumption. This approach allows us to achieve millisecond-level precision even with an inexpensive camera operating at a relatively low frame rate. One of our objectives was to keep the system cost-effective and broadly accessible, and this simplification supports that goal. While the experimental waves are not perfectly planar, this approximation enables a stable and tractable closed-loop system using accessible materials.

Our approach was first validated using projected waves, which provide controlled and reproducible patterns and are commonly used in our group to mimic cardiac wave propagation. As shown in Results (Section A) and Fig. 7, this setup achieves about millisecond-level precision. We then applied the same framework to real tissue in Fig. 8, demonstrating that the system remains functional and capable of influencing tissue dynamics despite increased variability and lower SNR.

We agree that more complex wave geometries and lower signal quality introduce additional challenges. However, the goal of this study was to demonstrate a minimal and accessible implementation of a closed-loop system. The framework itself is not limited to planar assumptions: it can be extended to incorporate more advanced detection methods, such as tracking wavefront shapes or estimating multi-directional propagation, as well as spatially patterned optogenetic stimulation (Fig. 5) in future studies.

To clarify these points, we have revised Fig. 8 to include additional snapshots and spatial scales, and we have updated the Discussion to better outline both the current assumptions and the potential extensions of the system. We have also added more details such as ROI size in the method section.

**12. The authors make the broad claim that the close-loop system successfully controls the state of the cell culture. I am not convinced yet that the authors provide sufficient evidence that the control has been successful.**

We agree that the wording in the original manuscript may have caused confusion. Our intention in this work is not to establish full model-based control of the cell culture in a strict sense, but rather to demonstrate that the system can reliably influence and redirect tissue dynamics through a closed-loop interaction. In Fig. 8, we show that the system is able to override the intrinsic pacing activity of the monolayer and reinitiate wave propagation from a different location, effectively altering the natural activation pattern. This demonstrates that the system can intervene in and modify the ongoing dynamics of the tissue.

However, we do not claim that a specific target state is being precisely controlled or maintained. The current study is designed as a proof of concept to show that such interaction is feasible and can be achieved in real time using an accessible setup. The framework allows the simulation to participate in the dynamics and provides a mechanism through which control strategies could be implemented. Future studies can build on this by defining specific target behaviors and systematically exploring how different parameter settings in the simulation lead to desired control outcomes. We have revised the manuscript to clarify this distinction and to avoid confusion about the level of control demonstrated in the present work.

**13. In the abstract, the authors state that they use physiologically relevant simulations. Why did the authors specifically choose a cellular automaton? The authors should substantiate their claim by showing model state and observation under various conditions and spatial-temporal dynamics.**

We thank the reviewer for raising this point regarding our choice of model. Cellular automata (CA) models have been widely used to represent excitable media and are known to reproduce key cardiac dynamics, including burst activity, spiral waves, and fibrillation [1, 2]. At the macroscopic level, these models capture both stable and unstable propagation patterns that are comparable to those observed in cardiac monolayers.

In this study, our choice of a CA model was also motivated by practical considerations. One of our main objectives was to develop a platform that is inexpensive and broadly accessible. CA models are computationally lightweight and can be efficiently implemented using WebGL through Abubu.js, allowing the system to run in real time on standard laptops with limited GPU resources. This makes the platform usable by a wide range of researchers and students without specialized hardware.

To clarify how model parameters influence the observed dynamics, we have revised Fig. 3 to include the corresponding simulation parameters and to illustrate how different parameter choices lead to different dynamical regimes. At the same time, we acknowledge that CA models are a simplified representation. To address this, we have made additional cardiac models available in our GitHub repository, including the Fenton-Karma model, and provided instructions for implementing other models available in Abubu.js [5]. This allows users to select a model that best matches their experimental needs and level of physiological detail.

**Reviewer #3:**

**The paper proposes a digital twin framework for optogenetic cardiac tissues. Closely following the definition of a digital twin, the approach is bidirectional: the computational model, based on cellular automata (CA), is informed by monolayer activation data, and the software can stimulate the tissue in a feedback-control loop. The authors address the challenge of real-time feedback, which is necessary for smooth control. They identify key bottlenecks, the main one being the computational cost of tissue-level cardiac simulations. This is addressed by adopting a cellular automaton implemented as a WebGL application, which can be efficiently queried via node.js. The presented experiments demonstrate the robustness of the approach compared to a fixed-time control loop.

 The paper is interesting and the overall setup is impressive, particularly because all described components (both hardware and software) are inexpensive and accessible. The manuscript is logically structured, the figures are mostly clear, and no typographical errors were found. Overall, the work appears to be a good fit for PLOS Computational Biology, and no critical flaws have been identified. However, there are several major issues and some minor suggestions that could improve the manuscript.

 Major issues
 -1. The introduction is logically structured, but it lacks a concrete motivating example. While reading, I found the approach exciting, yet I struggled to identify a clear and compelling application scenario. The example provided in the Results section (B) is not entirely clear. Figure 1 is also very conceptual and somewhat generic, and therefore not very helpful. For instance, why is the CA represented on a circular domain while the tissue is shown as a square? What do the arrows represent, current injection? stimulation? Additionally, Figure 8 does not sufficiently clarify the practical application.**

We thank the reviewer for highlighting the need for further clarification and for pointing out where the presentation could be improved. This comment helped us realize that the purpose and application of the system were not communicated clearly enough in the original version. A key source of confusion was our use of the term ‘digital twin’. While the system could, in principle, be extended in that direction, its current purpose is more accurately described as a coupled cardiac system, where a computational model interacts with and extends tissue dynamics in a closed loop. We have revised the manuscript accordingly to better reflect this distinction.

The main application we aim to address is the study of reentrant circuits (i.e., a spiral wave circulating around an unexcitable region), which are central to many cardiac arrhythmias. In traditional experimental settings, reentrant loops are either formed entirely within the tissue [6] or partially approximated using fixed-delay stimulation protocols [7], where a fixed delay replaces part of the circuit. In our approach, we replace part of this loop with a computational simulation: cardiac waves propagate through the tissue for part of the cycle and through the simulation for the remainder. This setup allows the user to control and modify the dynamics of the reentrant pathway through adjustable simulation parameters, while maintaining interaction with real tissue. The goal of this work is to demonstrate that such a system can be implemented in a simple and accessible way, and that it can influence tissue dynamics in real time.

In this context, Results Section B and Fig. 8 are intended as a proof of concept. We show that the system can override the intrinsic pacing of the monolayer and impose a new activation pattern, including the emergence of alternans by analyzing the reentrant periods, demonstrating that the coupled loop is functional. We have revised the text around these figures to better explain this application and to make the experimental outcome clearer.

We have also revised Fig. 1 to better illustrate the underlying concept. The circular domain was originally used as a schematic representation of a reentrant circuit, while the square domain represented a more practical and simplified implementation using a monolayer and a rectangular simulation grid. We agree that this distinction was not sufficiently clear. In the revised figure, we more explicitly show how the coupled system forms a reentrant loop. We have also updated Fig. 8 to better represent the system, where the arrows indicate the direction of wave propagation. In addition, we now clarify in the method the simulation geometry can be adapted by the user depending on the experimental design. Overall, we have revised the Introduction, figures, and figure descriptions to provide a more concrete application scenario and to improve clarity throughout.

**-2. It is not clear how the digital twin is calibrated to achieve one-to-one fidelity with the experimental tissue. If such strict fidelity is not required, this should be explicitly stated. Otherwise, the authors should better explain how they plan to achieve it, given the structural and dynamical constraints of the CA model.**

As explained in the previous response, our objective is to recreate a reentrant circuit in which half of the circuit is implemented in a digital simulation and the other half in cardiac culture, with the two systems coupled in a closed feedback loop. Although our system is capable of being tuned to replicate the monolayer as a digital twin, we now refer to it as a coupled cardiac system to address the reviewers valid concerns. In this framework, the cardiac tissue is only updated at the end of the loop through optogenetic stimulation, and therefore there is no requirement to achieve real-time, one-to-one fidelity between the simulation and the experimental tissue. We have revised the manuscript to clarify this point and avoid further misunderstanding.

**-3.The second example in the Results section, particularly Figure 8, is not sufficiently clear. Could the authors better elaborate on its aim and the corresponding results? My understanding is that an incoming wave is detected by the system (perhaps indicated by the rectangles), the CA model attempts to replicate this wave (although the similarity in the figure is not very evident), and then the system decides where to pace, as shown in the lower panels. Is this interpretation correct? What exactly does the bottom plot represent?**

We agree with the reviewer that our original Fig. 8 was not clear. As noted earlier, we have revised the terminology from digital twin to coupled cardiac system to better reflect the role of the simulation. Figure 8 is intended to illustrate this coupled system, where the simulation interacts with the cardiac monolayer to form a closed loop. More specifically, the experiment represents a reentrant circuit in which part of the loop is implemented in the tissue and the other part in the simulation.

In this setup, the cardiac wave is detected by the camera at two regions of interest (ROIs). By measuring the time difference between these ROIs, the system estimates the conduction velocity and predicts when the wave will reach the upper boundary of the tissue. Then, the simulation generates the second half of the loop, where a wave propagates from the top to the bottom of the simulated domain. Once this simulated propagation is completed, the system calculates the total loop timing and triggers the LEDs to stimulate the tissue at the appropriate moment, initiating the next cycle. The bottom plot represents the reentrant period across successive cycles. In our specific setup, we observe alternans behavior, where the cycle length alternates between two values (period-2 dynamics). To address the lack of clarity, we have revised Fig. 8 to include additional snapshots of the loop and updated the description in the manuscript to better explain each step of the process.

**-4. The CA model described in the Supplement does not appear very flexible in terms of tuning conduction velocity and other electrophysiological parameters. I understand the trade-off with computational speed; however, it would be helpful to clarify whether further adaptability is possible within this framework or whether the authors plan to move toward a different model in the future. For example, the group has strong expertise in real-time reaction-diffusion models. How far are we from achieving real-time performance with such models?**

We agree with the reviewer that the CA model has many limitations. Our goal is to develop a coupled cardiac system that is adaptable to a wide range of experimental settings. Although the CA is a phenomenological model we chose it for two main reasons. First, it has established relevance for cardiac dynamics, as previous studies have shown that CA models can reproduce key macroscopic behaviors of excitable media, including spiral waves and fibrillation [1, 2]. Second, it is computationally lightweight and can run in real time using WebGL on standard laptops with limited GPU resources. This was an important consideration, as one of our objectives is to make the system accessible to a broad range of research contexts.

At the same time, the framework is not restricted to the CA model. To address this concern, we have made additional cardiac models available through our GitHub repository, including the Fenton-Karma model, and provided instructions on how to integrate other models implemented in Abubu.js [5]. This allows users to select models with different levels of physiological detail and to tune parameters such as ion conductance or diffusion coefficients according to their experimental needs. Regarding real-time performance of more detailed reaction–diffusion models, this remains an active area of development. With WebGL, such models can achieve real-time performance in 2D, and even 3D, given sufficient GPU resources [5, 8]. However, our main objective in this work was to design a system that can be implemented on standard office laptops. We have revised the manuscript to better clarify this flexibility and the rationale behind our model choice.

**-5. Some parts of the Introduction, Discussion and Conclusion are repetitive. The overall readability could be improved by making certain sentences more precise and better contextualised, and by reducing redundancy.**

We thank the reviewer for this helpful comment on the readability of the manuscript. We have revised the Introduction, Discussion, and Conclusion to improve clarity and reduce repetition. In particular, we replaced the term digital twin with coupled cardiac system to better reflect the purpose of the work, and we revised several sections to make the arguments more precise and the overall flow smoother.  **Minor issues
 -6. It may be worth mentioning alternative approaches to achieving real-time performance, such as surrogate modelling or operator learning. In relatively simple geometries (e.g., a square domain), such techniques already exist and perform reasonably well in specific settings. While they may not yet match the efficiency of CA, they are likely to improve significantly, especially on hardware specifically designed for neural networks (not only TPUs). A brief discussion would help contextualise the chosen approach.**

We agree that surrogate modeling and operator learning are promising techniques, particularly as hardware designed for neural networks continues to improve. In our case, the simulation is implemented using WebGL, specifically through the Abubu.js library, which allows the model to run in real time, or even faster than the intrinsic pace of the tissue, on standard hardware. The choice of a cellular automaton (CA) model was mainly driven by our objective to make the system adaptable and accessible with minimal computational requirements. By adjusting the number of simulation steps through the simulation’s user interface, the effective simulation speed can be increased significantly. At the same time, more powerful GPUs commonly available in many labs can support more detailed models, such as OVVR, within the same framework, in real-time or faster.

A key advantage of this approach is flexibility. The WebGL implementation allows the simulation domain to be easily modified in real time. For example, users can interactively define unexcitable regions by shift-clicking on the canvas, or restore excitability using control-click, and reshape the domain directly according to experimental needs. This makes it straightforward to design and test different reentrant pathways or spatial configurations without additional preprocessing or model retraining.

In contrast, surrogate and operator learning approaches typically require a training dataset, careful model selection, and additional computational resources for training and validation. While these methods offer increased flexibility and the potential for higher physiological fidelity, they introduce an additional layer of complexity that is not necessary for demonstrating the core functionality of the coupled system presented here. We have added a brief discussion in the revised manuscript to acknowledge these approaches and to better position our method within the broader set of available techniques. We thank the reviewer for bringing up these alternative approaches, which are indeed relevant when considering real-time performance in computational models.

**- 7. The extension to 3D is likely more challenging than implied. While a 2D surface may represent a reasonable intermediate step, full 3D modelling presents significant challenges, not only computationally, but also technologically. Surface optical mapping likely reflects an integrated optical signal from the entire 3D tissue thickness. A more nuanced discussion of these limitations would strengthen the manuscript.**

We agree with the reviewer’s concerns regarding the challenges associated with extending the system to 3D. From a computational perspective, 3D simulations are feasible within our framework. As shown in Kaboudian et al. [5, 8], WebGL-based implementations can support real-time 3D cardiac models. In our own work, we have also implemented a 3D cellular automaton representation of the heart (represented in the figure below), where activation can be interactively induced at different locations within the volume. More complex stimulation patterns can similarly be defined within the model. While these simulations are achievable, they do require more GPU resources than typically available on low-end hardware, though they remain within reach of standard GPUs commonly found in many laboratories.

The main challenges arise on the experimental side. As the reviewer noted, optical mapping from the surface captures an integrated signal across tissue depth, making it difficult to resolve true three-dimensional wave dynamics. In addition, recording activity within the depth of the tissue is not accessible with our current camera-based approach. For a closed-loop system, targeting a specific region of interest on the surface may be sufficient for certain applications, but extending this to full 3D monitoring would require more advanced imaging techniques.

Similarly, for actuation, surface stimulation using LEDs is straightforward and compatible with the current setup. However, targeting specific regions within the depth of the tissue is more challenging. While approaches such as inserting optical probes could, in principle, address this, they introduce additional complications, including potential damage to the tissue. We have revised the manuscript to better reflect these distinctions, clarifying that while 3D simulation is computationally feasible with sufficient resources, the primary limitations in extending the full closed-loop system to 3D are currently experimental and technological.

**-8. In the Introduction, the OVVR model is mentioned before being defined.**

Fig. 1. 3D cellular automaton model representing a cardiac wave traveling within the tissue of a 3D heart. The color bar demonstrates cell activity levels, and waves propagate in the direction of the red color.

The manuscript was corrected to address this issue.

**-9. There is some repetition in the Methods sections B, C, and D regarding the code and repository URL. Since the URL is always the same, it could be mentioned once and subsequently referenced (e.g., to specific folders or files). Some of the descriptive text in these sections is also similar and could be streamlined.**

The manuscript was corrected to address the above issue.

**-10 Since the experiments are performed on iPSC-derived cells, are the FK or OVVR models appropriate choices for simulating their electrophysiology? A brief justification would be useful.**

In this study, our primary objective was to develop a coupled cardiac system that can operate in real time using accessible and inexpensive computational resources, requiring least GPU power. For this reason, we used a cellular automaton (CA) model, which provides a simple and computationally efficient representation of excitable media. As shown in previous work [1, 2, 9], CA models are capable of reproducing key macroscopic cardiac dynamics, including wave propagation, spiral waves, and fibrillation, making them suitable for demonstrating the functionality of the coupled system.

At the same time, the framework is not limited to the CA model. To address model-specific requirements, we have made additional models available in our GitHub repository, including the Fenton-Karma model, and provided instructions for integrating other models implemented in WebGL [5]. This allows users to select and tune models that are more appropriate for their specific experimental system. Our platform is flexible and can be adapted according to the experimental requirement. In this sense, the present work focuses on establishing the platform and its real-time interaction capabilities, while leaving model selection and physiological calibration to future studies tailored to specific biological questions.
 **- 11. Figure 1 in the Appendix does not appear to be properly scaled (0.05 compared to 1 on the y-axis).**

The required adjustment was applied to Fig 1 in the supplementary materials.

**References**

1.	Bub G, Shrier A, Glass L. Spiral wave generation in heterogeneous excitable media. Phys Rev Lett. 2002;88(5):058101. doi: 10.1103/PhysRevLett.88.058101.

2.	Bub G, Shrier A, Glass L. Global Organization of Dynamics in Oscillatory Heterogeneous Excitable Media. Phys Rev Lett. 2005;94(2):028105. doi: 10.1103/PhysRevLett.94.028105.

3.	Sala L, van Meer BJ, Tertoolen LGJ, Bakkers J, Bellin M, Davis RP, et al. MUSCLEMOTION. Circulation Research. 2018;122(3):e5-e16. doi: doi:10.1161/CIRCRESAHA.117.312067.

4.	Liu W, Han JL, Tomek J, Bub G, Entcheva E. Simultaneous Widefield Voltage and Dye-Free Optical Mapping Quantifies Electromechanical Waves in Human Induced Pluripotent Stem Cell-Derived Cardiomyocytes. ACS Photonics. 2023;10(4):1070-83. doi: 10.1021/acsphotonics.2c01644.

5.	Kaboudian A, Cherry EM, Fenton FH. Large-scale Interactive Numerical Experiments of Chaos, Solitons and Fractals in Real Time via GPU in a Web Browser. Chaos Solitons Fractals. 2019;121:6-29. Epub 20190216. doi: 10.1016/j.chaos.2019.01.005. PubMed PMID: 34764627; PubMed Central PMCID: PMCPMC8580290.

6.	González H, Nagai Y, Bub G, Glass L, Shrier A. Reentrant waves in a ring of embryonic chick ventricular cells imaged with a Ca2+ sensitive dye. BioSystems. 2003;71(1):71-80. doi: <https://doi.org/10.1016/S0303-2647(03)00111-4>.

7.	Scardigli M, Müllenbroich C, Margoni E, Cannazzaro S, Crocini C, Ferrantini C, et al. Real-time optical manipulation of cardiac conduction in intact hearts. J Physiol. 2018;596(17):3841-58. Epub 20180807. doi: 10.1113/jp276283. PubMed PMID: 29989169; PubMed Central PMCID: PMCPMC6117584.

8.	Kaboudian A, Cherry EM, Fenton FH. Real-time interactive simulations of large-scale systems on personal computers and cell phones: Toward patient-specific heart modeling and other applications. Science Advances. 2019;5(3):eaav6019. doi: doi:10.1126/sciadv.aav6019.

9.	Bub G, Shrier A. Propagation through heterogeneous substrates in simple excitable media models. Chaos. 2002;12(3):747-53. doi: 10.1063/1.1502481.
