# Supplementary material for: Real-time GPU-accelerated coupled cardiac system: Integrating bidirectional interactions between living optogenetic monolayers and computational simulations
Source: PLoS Comput Biol. 2026 Aug 3;22(8):e1014590. doi: 10.1371/journal.pcbi.1014590 (PMC13450847; doi:10.1371/journal.pcbi.1014590)
Supplement: Peer review history

# Peer review: Decision Letter 0

**DOI:** 10.1371/journal.pcbi.1014590.r001

**Authors:** Marc Birtwistle, Anna Grosberg

4 Mar 2026

PCOMPBIOL-D-26-00123

Real-Time GPU-Accelerated Digital Heart Twin: Integrating Bidirectional Interactions Between Living Optogenetic Monolayers and Computational Simulations

PLOS Computational Biology

Dear Dr. Valibeigi,

Thank you for submitting your manuscript to PLOS Computational Biology. After careful consideration, we feel that it has merit but does not fully meet PLOS Computational Biology's publication criteria as it currently stands. Therefore, we invite you to submit a revised version of the manuscript that addresses the points raised during the review process. A concern was raised that the work in this paper does not represent a true digital twin framework and the hardware innovations might be better suited to a different journal. If you decide to resubmit, please ensure that you have fully addressed these concerns as well as the other detailed in the reviews.

Please submit your revised manuscript by May 04 2026 11:59PM. If you will need more time than this to complete your revisions, please reply to this message or contact the journal office at ploscompbiol@plos.org. When you're ready to submit your revision, log on to https://www.editorialmanager.com/pcompbiol/ and select the 'Submissions Needing Revision' folder to locate your manuscript file.

Please include the following items when submitting your revised manuscript:

* A letter that responds to each point raised by the editor and reviewer(s). You should upload this letter as a separate file labeled 'Response to Reviewers'. This file does not need to include responses to formatting updates and technical items listed in the 'Journal Requirements' section below.

* A marked-up copy of your manuscript that highlights changes made to the original version. You should upload this as a separate file labeled 'Revised Manuscript with Track Changes'.

* An unmarked version of your revised paper without tracked changes. You should upload this as a separate file labeled 'Manuscript'.

If you would like to make changes to your financial disclosure, competing interests statement, or data availability statement, please make these updates within the submission form at the time of resubmission. Guidelines for resubmitting your figure files are available below the reviewer comments at the end of this letter

We look forward to receiving your revised manuscript.

Kind regards,

Anna Grosberg, Ph.D.

Academic Editor

PLOS Computational Biology

Marc Birtwistle

Section Editor

PLOS Computational Biology

**Journal Requirements:**

1) Please provide an Author Summary. This should appear in your manuscript between the Abstract (if applicable) and the Introduction, and should be 150-200 words long. The aim should be to make your findings accessible to a wide audience that includes both scientists and non-scientists. Sample summaries can be found on our website under Submission Guidelines:

https://journals.plos.org/ploscompbiol/s/submission-guidelines#loc-parts-of-a-submission

2) Please upload all main figures as separate Figure files in .tif or .eps format. For more information about how to convert and format your figure files please see our guidelines:

https://journals.plos.org/ploscompbiol/s/figures

3) We have noticed that you have uploaded Supporting Information files, but you have not included a list of legends. Please add a full list of legends for your Supporting Information files after the references list.

4) Some material included in your submission may be copyrighted. According to PLOSu2019s copyright policy, authors who use figures or other material (e.g., graphics, clipart, maps) from another author or copyright holder must demonstrate or obtain permission to publish this material under the Creative Commons Attribution 4.0 International (CC BY 4.0) License used by PLOS journals. Please closely review the details of PLOSu2019s copyright requirements here: PLOS Licenses and Copyright. If you need to request permissions from a copyright holder, you may use PLOS's Copyright Content Permission form.

Please respond directly to this email and provide any known details concerning your material's license terms and permissions required for reuse, even if you have not yet obtained copyright permissions or are unsure of your material's copyright compatibility. Once you have responded and addressed all other outstanding technical requirements, you may resubmit your manuscript within Editorial Manager.

Potential Copyright Issues:

- Figures 1, 2, 5, 6, and 8: Please confirm whether you drew the images / clip-art within the figure panels by hand. If you did not draw the images, please provide (a) a link to the source of the images or icons and their license / terms of use; or (b) written permission from the copyright holder to publish the images or icons under our CC BY 4.0 license. Alternatively, you may replace the images with open source alternatives. See these open source resources you may use to replace images / clip-art:

- https://commons.wikimedia.org

- https://openclipart.org/.

5) Please amend your detailed Financial Disclosure statement. This is published with the article. It must therefore be completed in full sentences and contain the exact wording you wish to be published.

- State the initials, alongside each funding source, of each author to receive each grant. For example: "This work was supported by the National Institutes of Health (####### to AM; ###### to CJ) and the National Science Foundation (###### to AM)."

- State what role the funders took in the study. If the funders had no role in your study, please state: "The funders had no role in study design, data collection and analysis, decision to publish, or preparation of the manuscript.".

If you did not receive any funding for this study, please simply state: u201cThe authors received no specific funding for this work.u201d

**Reviewers' comments:**

Reviewer's Responses to Questions

**Comments to the Authors:**

**Please note here if the review is uploaded as an attachment.**

Reviewer #1: The paper descries a highly novel and innovative experimental – simulation hybrid system that would allow for sophisticated experiments. I have some general concerns that would be good to address.

The authors make a number of statements which may be true, but are not supported by evidence. For example, they could have created a digital twin of the simulation system to explore how delays, resolutions, or other differences impact the results. At present, they claim the system needs to be fast and then make it faster. This means we are told how accurate the system is, but no quantitative metric or formal evaluation is defined to demonstrate that it is fast enough.

The paper describes a model–experimental coupling, but it does not describe a digital twin. A digital twin should represent the experiment, not act as an additional pathway within it. The authors should rename and rephrase this element of the paper to avoid confusion.

The results section does not show how well the digital twin replicates the experimental set-up. This critical result is therefore absent from the paper. The authors do not define target accuracies or tolerances for the twin to be effective, making it impossible to statistically evaluate the twin.

No biological insight is presented. The authors may want to consider whether this work is more suitable for an IEEE journal rather than a computational biology journal.

Finally, the digital twin was not tuned to represent the experiment. If this tuning was performed, substantially more detail should be provided.

Reviewer #2: In the manuscript “Real-Time GPU-Accelerated Digital Heart Twin: Integrating Bidirectional Interactions Between Living Optogenetic Monolayers and Computational Simulations” by Younes Valibeigi and co-workers addresses a relevant research topic of potentially broad interest.

A digital twin is a dynamic, virtual replica of a physical system that is continuously updated with real-time data from its physical counterpart. Sensor Data from the physical system is fed into the digital twin in real-time to reflect the current state.

In the system investigated in this manuscript, the physical system is a two-dimensional cardiac optogenetic cell culture. The state of the system is obtained by optical sensors (digital camera), that measure the mechanical deformation of excitable cells. Actuators are multiple LEDs in an array that can activate optogenetically modified channels. The digital twin is implemented for simplicity as a cellular automaton.

While the research question is highly relevant, I have some concerns regarding the methods and results. I suggest a major revision of the manuscript.

Additionally, several variants of the digital twin methodology have already been published, most recently for example https://lnkd.in/e9GYsTmW Therefore, the authors should further highlight their innovation.

From the data presented in the manuscript, it remains unclear that a digital twin has been implemented, i.e., a virtual replica of the observed biological system. To what extend does the dynamics of the numerical simulation represent the observation? The observed quantity is the mechanical contraction of the cells. However, the model describes a generic excitable medium. How do the simulation relates to the properties of the cell culture, for example, geometry of the culture, or dynamical properties such as the propagation of electrical or mechanical waves, restitution dynamics for APD and CV etc.? If Fig. 3 represents possible states of the digital twin, how do these states quantitively compare to observed wave dynamics in the cell culture (for example with respect to spatial-temporal scales, wave lengths etc.) shown in Fig. 8?

The dynamics of cell cultures depend on several parameters, such as time of cultivation, cell density, homogeneity etc. and are expected to vary from experiment to experiment. How are these parameters reflected in the model and how are parameters (such as diffusion constant/tensor) estimated from the observation? Again, a direct comparison of model state and observations (e.g. time series of model output and observation) could be helpful to answer the question. Furthermore, to what extend can the model predict the dynamics of the system?

More specifically, Fig. 8B shows observed waves traveling upward in the cell culture image. The simulation shows a wave (with scale bar missing) apparently showing wave propagation in the simulation. The simulation is shown a wave (colorbar missing). Is this simulation snapshot supposed to show a replica of the observed state? If so, how? Also, additional data that goes beyond an anecdotal example should be provided.

Furthermore, it is not clear that the Fig. 8 confirms that the digital twin can support model-based control, as claimed by the authors. The authors should elaborate how data shown in this figure demonstrate successful control? If control is shown, which is the desired target dynamics?

The SNR of these waves appears to be rather low in the observed wave patterns shown in Fig. 8. Low SNR requires to average wave dynamics over large regions of interests. Without spatial scales provided (wave length of excitation wave, size of roi etc.), it remains unclear what the limitations are. In this regard, the conduction velocity measurement assumes planar waves and estimates only one velocity component. The waves in the experiments, however, appear not be planar (see Fig. 8). If remains unclear how, given these significant limitations, spatial-temporal control of complex patterns can be achieved.

The authors make the broad claim that the close-loop system successfully controls the state of the cell culture. I am not convinced yet that the authors provide sufficient evidence that the control has been successful.

In the abstract, the authors state that they use physiologically relevant simulations. Why did the authors specifically choose a cellular automaton? The authors should substantiate their claim by showing model state and observation under various conditions and spatial-temporal dynamics.

Reviewer #3: The paper proposes a digital twin framework for optogenetic cardiac tissues. Closely following the definition of a digital twin, the approach is bidirectional: the computational model, based on cellular automata (CA), is informed by monolayer activation data, and the software can stimulate the tissue in a feedback-control loop. The authors address the challenge of real-time feedback, which is necessary for smooth control. They identify key bottlenecks, the main one being the computational cost of tissue-level cardiac simulations. This is addressed by adopting a cellular automaton implemented as a WebGL application, which can be efficiently queried via node.js. The presented experiments demonstrate the robustness of the approach compared to a fixed-time control loop.

The paper is interesting and the overall setup is impressive, particularly because all described components (both hardware and software) are inexpensive and accessible. The manuscript is logically structured, the figures are mostly clear, and no typographical errors were found. Overall, the work appears to be a good fit for PLOS Computational Biology, and no critical flaws have been identified. However, there are several major issues and some minor suggestions that could improve the manuscript.

Major issues

- The introduction is logically structured, but it lacks a concrete motivating example. While reading, I found the approach exciting, yet I struggled to identify a clear and compelling application scenario. The example provided in the Results section (B) is not entirely clear. Figure 1 is also very conceptual and somewhat generic, and therefore not very helpful. For instance, why is the CA represented on a circular domain while the tissue is shown as a square? What do the arrows represent, current injection? stimulation? Additionally, Figure 8 does not sufficiently clarify the practical application.

- It is not clear how the digital twin is calibrated to achieve one-to-one fidelity with the experimental tissue. If such strict fidelity is not required, this should be explicitly stated. Otherwise, the authors should better explain how they plan to achieve it, given the structural and dynamical constraints of the CA model.

- The second example in the Results section, particularly Figure 8, is not sufficiently clear. Could the authors better elaborate on its aim and the corresponding results? My understanding is that an incoming wave is detected by the system (perhaps indicated by the rectangles), the CA model attempts to replicate this wave (although the similarity in the figure is not very evident), and then the system decides where to pace, as shown in the lower panels. Is this interpretation correct? What exactly does the bottom plot represent?

- The CA model described in the Supplement does not appear very flexible in terms of tuning conduction velocity and other electrophysiological parameters. I understand the trade-off with computational speed; however, it would be helpful to clarify whether further adaptability is possible within this framework or whether the authors plan to move toward a different model in the future. For example, the group has strong expertise in real-time reaction-diffusion models. How far are we from achieving real-time performance with such models?

- Some parts of the Introduction, Discussion and Conclusion are repetitive. The overall readability could be improved by making certain sentences more precise and better contextualised, and by reducing redundancy.

Minor issues

- It may be worth mentioning alternative approaches to achieving real-time performance, such as surrogate modelling or operator learning. In relatively simple geometries (e.g., a square domain), such techniques already exist and perform reasonably well in specific settings. While they may not yet match the efficiency of CA, they are likely to improve significantly, especially on hardware specifically designed for neural networks (not only TPUs). A brief discussion would help contextualise the chosen approach.

- The extension to 3D is likely more challenging than implied. While a 2D surface may represent a reasonable intermediate step, full 3D modelling presents significant challenges, not only computationally, but also technologically. Surface optical mapping likely reflects an integrated optical signal from the entire 3D tissue thickness. A more nuanced discussion of these limitations would strengthen the manuscript.

- In the Introduction, the OVVR model is mentioned before being defined.

- There is some repetition in the Methods sections B, C, and D regarding the code and repository URL. Since the URL is always the same, it could be mentioned once and subsequently referenced (e.g., to specific folders or files). Some of the descriptive text in these sections is also similar and could be streamlined.

- Since the experiments are performed on iPSC-derived cells, are the FK or OVVR models appropriate choices for simulating their electrophysiology? A brief justification would be useful.

- Figure 1 in the Appendix does not appear to be properly scaled (0.05 compared to 1 on the y-axis).

**********

**Have the authors made all data and (if applicable) computational code underlying the findings in their manuscript fully available?**

The PLOS Data policy requires authors to make all data and code underlying the findings described in their manuscript fully available without restriction, with rare exception (please refer to the Data Availability Statement in the manuscript PDF file). The data and code should be provided as part of the manuscript or its supporting information, or deposited to a public repository. For example, in addition to summary statistics, the data points behind means, medians and variance measures should be available. If there are restrictions on publicly sharing data or code —e.g. participant privacy or use of data from a third party—those must be specified.

Reviewer #1: Yes

Reviewer #2: Yes

Reviewer #3: Yes

**********

PLOS authors have the option to publish the peer review history of their article (what does this mean?). If published, this will include your full peer review and any attached files.

If you choose “no”, your identity will remain anonymous but your review may still be made public.

**Do you want your identity to be public for this peer review?**  For information about this choice, including consent withdrawal, please see our Privacy Policy.

Reviewer #1: No

Reviewer #2: No

Reviewer #3: No

[NOTE: If reviewer comments were submitted as an attachment file, they will be attached to this email and accessible via the submission site. Please log into your account, locate the manuscript record, and check for the action link "View Attachments". If this link does not appear, there are no attachment files.]

**Figure resubmission:**

While revising your submission, we strongly recommend that you use PLOS’s NAAS tool (https://ngplosjournals.pagemajik.ai/artanalysis) to test your figure files. NAAS can convert your figure files to the TIFF file type and meet basic requirements (such as print size, resolution), or provide you with a report on issues that do not meet our requirements and that NAAS cannot fix.

After uploading your figures to PLOS’s NAAS tool - https://ngplosjournals.pagemajik.ai/artanalysis, NAAS will process the files provided and display the results in the "Uploaded Files" section of the page as the processing is complete. If the uploaded figures meet our requirements (or NAAS is able to fix the files to meet our requirements), the figure will be marked as "fixed" above. If NAAS is unable to fix the files, a red "failed" label will appear above. When NAAS has confirmed that the figure files meet our requirements, please download the file via the download option, and include these NAAS processed figure files when submitting your revised manuscript.

**Reproducibility:**

To enhance the reproducibility of your results, we recommend that authors of applicable studies deposit laboratory protocols in protocols.io, where a protocol can be assigned its own identifier (DOI) such that it can be cited independently in the future. Additionally, PLOS ONE offers an option to publish peer-reviewed clinical study protocols. Read more information on sharing protocols at https://plos.org/protocols?utm_medium=editorial-email&utm_source=authorletters&utm_campaign=protocols

# Author comment: Author response to Decision Letter 1

**DOI:** 10.1371/journal.pcbi.1014590.r002

7 May 2026

Attachment Submitted filename: V10_ResponseToReviewers.docx

# Peer review: Decision Letter 1

**DOI:** 10.1371/journal.pcbi.1014590.r003

**Authors:** Marc Birtwistle, Anna Grosberg

19 Jun 2026

PCOMPBIOL-D-26-00123R1

Real-Time GPU-Accelerated Coupled Cardiac System: Integrating Bidirectional Interactions Between Living Optogenetic Monolayers and Computational Simulations

PLOS Computational Biology

Dear Dr. Valibeigi,

Thank you for submitting your manuscript to PLOS Computational Biology. After careful consideration, we feel that it has merit but does not fully meet PLOS Computational Biology's publication criteria as it currently stands. The reviewers were happy with the technical state of the work, but please provide a discussion of the biological insights afforded by this paper. Therefore, we invite you to submit a revised version of the manuscript that addresses the points raised during the review process.

Please submit your revised manuscript by Aug 19 2026 11:59PM. If you will need more time than this to complete your revisions, please reply to this message or contact the journal office at ploscompbiol@plos.org. When you're ready to submit your revision, log on to https://www.editorialmanager.com/pcompbiol/ and select the 'Submissions Needing Revision' folder to locate your manuscript file.

Please include the following items when submitting your revised manuscript:

* A letter that responds to each point raised by the editor and reviewer(s). You should upload this letter as a separate file labeled 'Response to Reviewers'. This file does not need to include responses to formatting updates and technical items listed in the 'Journal Requirements' section below.

* A marked-up copy of your manuscript that highlights changes made to the original version. You should upload this as a separate file labeled 'Revised Manuscript with Track Changes'.

* An unmarked version of your revised paper without tracked changes. You should upload this as a separate file labeled 'Manuscript'.

If you would like to make changes to your financial disclosure, competing interests statement, or data availability statement, please make these updates within the submission form at the time of resubmission. Guidelines for resubmitting your figure files are available below the reviewer comments at the end of this letter.

As the corresponding author, your ORCID iD is verified in the submission system and will appear in the published article. PLOS supports the use of ORCID, and we encourage all coauthors to register for an ORCID iD and use it as well. Please encourage your coauthors to verify their ORCID iD within the submission system before final acceptance, as unverified ORCID iDs will not appear in the published article. Only the individual author can complete the verification step; PLOS staff cannot verify ORCID iDs on behalf of authors.

We look forward to receiving your revised manuscript.

Kind regards,

Anna Grosberg, Ph.D.

Academic Editor

PLOS Computational Biology

Marc Birtwistle

Section Editor

PLOS Computational Biology

**Additional Editor Comments (if provided):**

**Reviewers' comments:**

Reviewer's Responses to Questions

**Comments to the Authors:**

**Please note here if the review is uploaded as an attachment.**

Reviewer #1: My concerns have broadly been addressed through clarification of the paper’s claims.

However, I would still have liked to see either an experiment that would have been difficult to perform without the simulator, or a clearer demonstration that the system provides new biological insight.

Reviewer #3: Thanks for addressing my comments.

**********

**Have the authors made all data and (if applicable) computational code underlying the findings in their manuscript fully available?**

The PLOS Data policy requires authors to make all data and code underlying the findings described in their manuscript fully available without restriction, with rare exception (please refer to the Data Availability Statement in the manuscript PDF file). The data and code should be provided as part of the manuscript or its supporting information, or deposited to a public repository. For example, in addition to summary statistics, the data points behind means, medians and variance measures should be available. If there are restrictions on publicly sharing data or code —e.g. participant privacy or use of data from a third party—those must be specified.

Reviewer #1: Yes

Reviewer #3: Yes

**********

PLOS authors have the option to publish the peer review history of their article (what does this mean?). If published, this will include your full peer review and any attached files.

If you choose “no”, your identity will remain anonymous but your review may still be made public.

**Do you want your identity to be public for this peer review?**  For information about this choice, including consent withdrawal, please see our Privacy Policy.

Reviewer #1: No

Reviewer #3: No

[NOTE: If reviewer comments were submitted as an attachment file, they will be attached to this email and accessible via the submission site. Please log into your account, locate the manuscript record, and check for the action link "View Attachments". If this link does not appear, there are no attachment files.]

**Figure resubmission:**

-->While revising your submission, we strongly recommend that you use PLOS’s NAAS tool (https://ngplosjournals.pagemajik.ai/artanalysis) to test your figure files. NAAS can convert your figure files to the TIFF file type and meet basic requirements (such as print size, resolution), or provide you with a report on issues that do not meet our requirements and that NAAS cannot fix.

After uploading your figures to PLOS’s NAAS tool - https://ngplosjournals.pagemajik.ai/artanalysis, NAAS will process the files provided and display the results in the "Uploaded Files" section of the page as the processing is complete. If the uploaded figures meet our requirements (or NAAS is able to fix the files to meet our requirements), the figure will be marked as "fixed" above. If NAAS is unable to fix the files, a red "failed" label will appear above. When NAAS has confirmed that the figure files meet our requirements, please download the file via the download option, and include these NAAS processed figure files when submitting your revised manuscript.

**Reproducibility:**

To enhance the reproducibility of your results, we recommend that authors of applicable studies deposit laboratory protocols in protocols.io, where a protocol can be assigned its own identifier (DOI) such that it can be cited independently in the future. Additionally, PLOS ONE offers an option to publish peer-reviewed clinical study protocols. Read more information on sharing protocols at https://plos.org/protocols?utm_medium=editorial-email&utm_source=authorletters&utm_campaign=protocols

# Author comment: Author response to Decision Letter 2

**DOI:** 10.1371/journal.pcbi.1014590.r004

14 Jul 2026

Attachment Submitted filename: V11_ResponseToReviewers.docx

# Editor report: Decision Letter 2

**DOI:** 10.1371/journal.pcbi.1014590.r005

**Authors:** Marc Birtwistle, Anna Grosberg

17 Jul 2026

Dear Valibeigi,

We are pleased to inform you that your manuscript 'Real-Time GPU-Accelerated Coupled Cardiac System: Integrating Bidirectional Interactions Between Living Optogenetic Monolayers and Computational Simulations' has been provisionally accepted for publication in PLOS Computational Biology.

Before your manuscript can be formally accepted you will need to complete some formatting changes, which you will receive in a follow up email. A member of our team will be in touch with a set of requests.

Please note that your manuscript will not be scheduled for publication until you have made the required changes, so a swift response is appreciated.

IMPORTANT: The editorial review process is now complete. PLOS will only permit corrections to spelling, formatting or significant scientific errors from this point onwards. Requests for major changes, or any which affect the scientific understanding of your work, will cause delays to the publication date of your manuscript.

Should you, your institution's press office or the journal office choose to press release your paper, you will automatically be opted out of early publication. We ask that you notify us now if you or your institution is planning to press release the article. All press must be co-ordinated with PLOS.

Thank you again for supporting Open Access publishing; we are looking forward to publishing your work in PLOS Computational Biology.

Best regards,

Anna Grosberg, Ph.D.

Academic Editor

PLOS Computational Biology

Marc Birtwistle

Section Editor

PLOS Computational Biology

***********************************************************

# Editor report: Acceptance letter

**DOI:** 10.1371/journal.pcbi.1014590.r006

**Authors:** Marc Birtwistle, Anna Grosberg

PCOMPBIOL-D-26-00123R2

Real-Time GPU-Accelerated Coupled Cardiac System: Integrating Bidirectional Interactions Between Living Optogenetic Monolayers and Computational Simulations

Dear Dr Valibeigi,

I am pleased to inform you that your manuscript has been formally accepted for publication in PLOS Computational Biology. Your manuscript is now with our production department and you will be notified of the publication date in due course.

The corresponding author will soon be receiving a typeset proof for review, to ensure errors have not been introduced during production. Please review the PDF proof of your manuscript carefully, as this is the last chance to correct any errors. Please note that major changes, or those which affect the scientific understanding of the work, will likely cause delays to the publication date of your manuscript.

Soon after your final files are uploaded, unless you have opted out, the early version of your manuscript will be published online. The date of the early version will be your article's publication date. The final article will be published to the same URL, and all versions of the paper will be accessible to readers.

For Research, Software, and Methods articles, you will receive an invoice from PLOS for your publication fee after your manuscript has reached the completed accept phase. If you receive an email requesting payment before acceptance or for any other service, this may be a phishing scheme. Learn how to identify phishing emails and protect your accounts at https://explore.plos.org/phishing.

Thank you again for supporting PLOS Computational Biology and open-access publishing. We are looking forward to publishing your work!

With kind regards,

Anitha Samidurai

PLOS Computational Biology | Carlyle House, Carlyle Road, Cambridge CB4 3DN | United Kingdom ploscompbiol@plos.org | Phone +44 (0) 1223-442824 | ploscompbiol.org | @PLOSCompBiol
